# An Improved Method for the Quaternization of Nicotinamide and Antifungal Activities of Its Derivatives

**DOI:** 10.3390/molecules24061001

**Published:** 2019-03-13

**Authors:** Tamara Siber, Valentina Bušić, Dora Zobundžija, Sunčica Roca, Dražen Vikić-Topić, Karolina Vrandečić, Dajana Gašo-Sokač

**Affiliations:** 1 Faculty of Agrobiotechnical Sciences, Josip Juraj Strossmayer University of Osijek, Vladimira Preloga 1, HR–31000 Osijek, Croatia; tamara.siber@pfos.hr (T.S.); kvrandecic@pfos.hr (K.V.); 2 Faculty of Food Technology, Josip Juraj Strossmayer University of Osijek, Kuhačeva 20, HR–31000 Osijek, Croatia; vbusic@ptfos.hr (V.B.); dzobundzija@ptfos.hr (D.Z.); 3 NMR Centre, Ruđer Bošković Institute, Bijenička cesta 54, HR–10000 Zagreb, Croatia; sroca@irb.hr (S.R.); vikic@irb.hr (D.V.-T.); 4 Department of Natural and Health Sciences, Juraj Dobrila University of Pula, Zagrebačka 30, HR–52100 Pula, Croatia

**Keywords:** quaternization, nicotinamide, substituted 2-bromoacetophenones, microwave synthesis, antifungal activity

## Abstract

The quaternization reactions of nicotinamide, with different electrophiles: methyl iodide and substituted 2-bromoacetophenones (4-Cl, 4-Br, 4-H, 4-CH_3_, 4-F, 4-OCH_3_, 4-Ph, 2-OCH_3_, 4-NO_2_) are reported. The preparations were carried out by conventional synthesis and under microwave irradiation in absolute ethanol and acetone. The synthesis performed by microwave dielectric heating significantly improved yield, up to 8 times, and shortened down the reaction time from *ca.* one day in conventional, to 10–20 min. The structures of the synthesized compounds were confirmed by IR, ^1^H- and ^13^C-NMR spectroscopy, mass spectrometry and elemental analysis. The compounds have been screened for antifungal activities against *Fusarium oxysporum*, *Fusarium culmorum*, *Macrophomina phaseolina* and *Sclerotinia sclerotiorum* at concentrations of 10 µg/mL and 100 µg/mL. Six compounds showed the strong inhibition of mycelium growth at a concentration of 10 µg/mL. All tested compounds revealed the great inhibitory activities against *S. sclerotiorum* at a concentration of 100 µg/mL.

## 1. Introduction

**Figure 1:**
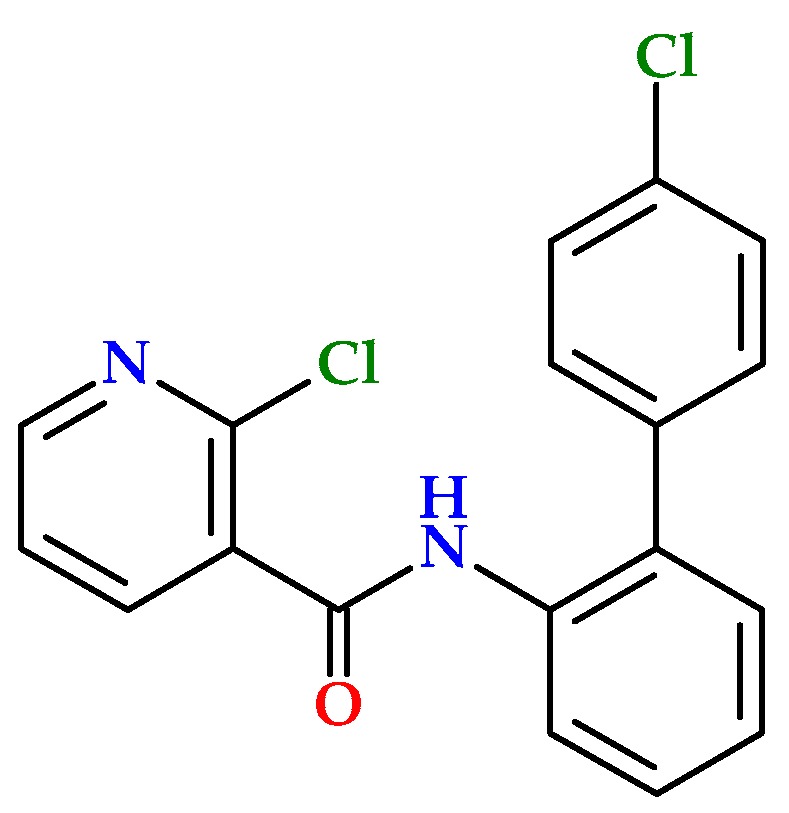
The structure of boscalid.

Nicotinamide is an important heterocyclic compound, which together with nicotinic acid, belongs to vitamin B3. Vitamin B3 is biosynthetically converted to nicotinamide adenine dinucleotide (NAD^+^), a versatile acceptor of hydride equivalents, to form the reduced dinucleotide, NADH. Nicotinamide is a water-soluble B complex vitamin which is naturally present in animal products, whole cereals and legumes [1]. Derivatives of nicotinamide have attracted much attention because of their antimicrobial [2,3,4,5,6], fungicidal [7,8,9,10,11], insecticidal [12,13] and herbicidal activity [14,15,16], plant growth regulator activity [17], cytotoxic properties [18,19], and antiangiogenesis activity [20]. Some nicotinamide derivatives, like boscalid, are used as commercial antifungicides (Figure 1). All fungicide synthesis studies revealed changes in the sub-structural unit of the amide backbone group of nicotinamide.

**Figure 2:**
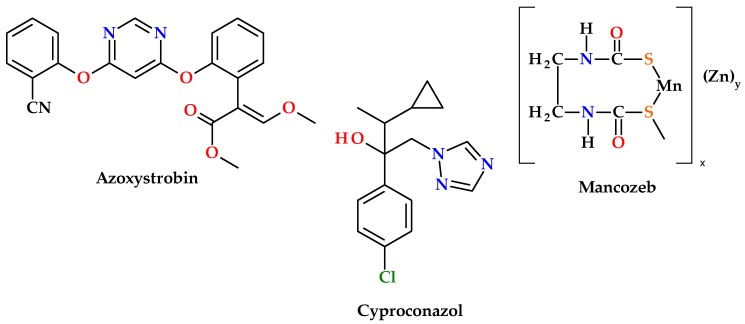
Some fungicides used in commercial products.

Related to our previous research [21,22] in which we carried out the quaternization of vitamin B6 derivatives with substituted 2-bromoacetophenones, in this study we investigated whether vitamin B3 is a good-suited nucleophile for the microwave (MW) assisted quaternization reaction with the same electrophiles. For this purpose, alkyl halides (methyl iodide) and substituted 2-bromoacetophenone have been used as electrophiles. New potential fungicidal molecules were prepared by using a simple and easily repeatable microwave assisted method. We investigated whether the derivatives with the quaternary nitrogen atom and the phenacyl unit are effective fungicides comparing to commercial agricultural ones. Fungicides are mainly toxic substances, so the goal of this work was to produce environmentally friendly fungicides, the derivatives of vitamin B3, which will be less toxic than commercial ones. In this research, fungicides from the group strobilurin and dithiocarbamates that have been approved for a specific culture of fungi in Croatia have been used as the standard (Figure 2). 

## 2. Results

### 2.1. Chemistry

**Scheme 1:**
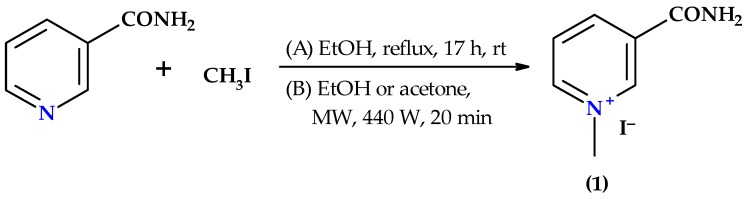
Conventional (A) and microwave irradiation (B) synthesis of 3-carbamoyl-1-methylpyridinium iodide (**1**).

**Scheme 2:**
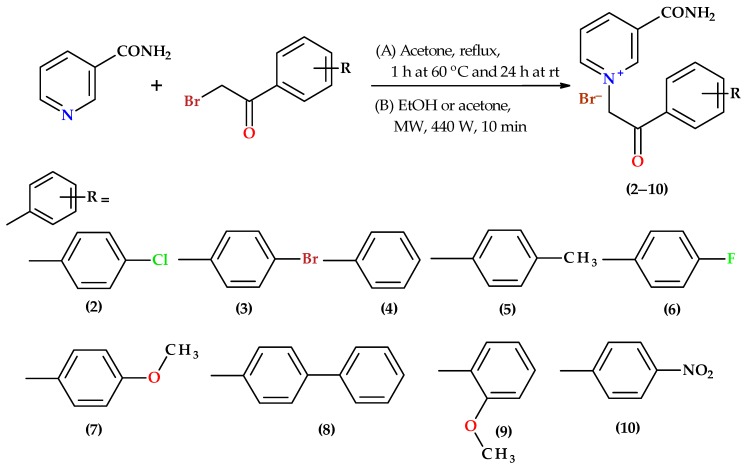
Conventional (A) and microwave irradiation (B) synthetic routes to nicotinamide derivatives with 2-bromoacetophenones (**2**–**10**).

Here we report the synthesis of ten (**1**–**10**) nicotinamide derivatives (Scheme 1 and Scheme 2).

Optimum conditions for performing microwave-assisted reactions were ascertained by carrying out reaction of nicotinamide and 2-bromoacetophenone (4). The results, summarized in Table 1, showed that the maximum yield of product (93%) was obtained by heating the sample at 440 W for 10 min.

The main disadvantages of the conventional method are a long reaction time and lower yields. Conventional synthesis of nicotinamide and methyl iodide (**1**) took place up to 17 h, and with the substituted 2-bromoacetophenone (**3**) up to 25 h. 

Differences between results obtained by conventional synthesis of quaternary salts of nicotinamide and by microwave-assisted method are shown in Table 2. The highest yield (78%) in the conventional method was achieved by quaternization of nicotinamide and methyl iodide **(1)**. Among the investigated substituted 2-bromoacetophenones, the highest yield (51%) was obtained with 2-brom-4-bromoacetophenone (**3**). The lowest yield was found for compound (**7**) (11%) and for (**5**) (18%). All other compounds (**2**, **6**, **8**–**10**) have yields between 31–39%. Zhuravlev et al. have synthesized nicotinamide salts (**4**) and (**7**) by conventional heating in anhydrous alcohol for 1 h, which gave rise to yield of 44% for compound (**4**), and 21% for compound (**7**) [23]. The same reactions were performed in our study by conventional method in acetone, giving yields of 25% and 11%, respectively.

The nucleophilic substitution of halogen in the α-haloketones (**3**) is considerably easy because of the greater electrophilicity of the carbocation adjacent to the acceptor carbonyl group. In nicotinamide the substituent decreases the nucleophilicity of nitrogen due to its negative inductive *–I* effect [σm(CONH_2_) = +0.28]. Consequently, the presence in the substrate molecule of a highly acceptor carbonyl group, as in the case of phenacyl bromide, leads to redistribution of electron density in the molecule, and the weakening of C–Br bond resulted in easier removal of the leaving group.

Significantly reducing reaction times from ca. 1 day in the conventional method to 10–20 min in the MW procedure is the big advantage of using microwave synthesis. Generally, all obtained yields in microwave synthesis are higher in ethanol than in acetone, except for (**8**) where it is the opposite, most probably due to the influence of the phenyl group, and for (**2**) and (**10**) where obtained yields are almost equal in both solvents. The highest yields in ethanol and acetone were achieved for (**1**) (>92%). We can assume that methyl iodide is the most reactive since the iodides are better leaving groups in nucleophilic substitution than bromides. Among substituted 2-bromoacetophenones, the highest yield in ethanol was obtained for (**4**) (93%), (**7**) (90%) and (**5**) (88%). Slightly lower yields were found for (**6**) (71%), (**2**) (67%) and (**3**) (64%), while the lowest yields were for (**8**–**10**), which are between 41% and 44%. The highest yield in acetone was obtained for (**8**) (80%), and only a little less was yielded for (**4**), (**5**) and (**7**) (73–76%). The yield for (**2**) was 65%, for (**3**) was 56%, and for (**6**) was 46%. The lowest yields in ethanol and acetone were obtained for (**9**), amounting 41% and 36%, respectively. This corresponds with steric hindrance of –OCH_3_ group in (**9**), in *ortho* position of 2-bromoacetophenone. The yield differences in used solvents can be attributed to the nature of the molecules. The highest yields in ethanol can be explained with the tangent *δ* value, which is less for ethanol than for acetone [24]. Ethanol is a high medium absorbing solvent (tan *δ* = 0.941), while acetone is a low medium one (tan *δ* = 0.054).

The chemical structures of investigated compounds were determined on the basis of spectral data analysis of IR, ^1^H- and ^13^C-NMR spectra in solution, elemental analysis and mass spectral analysis. All compounds show the absorption bands for *ν*N–H, *ν*NH_2_, *ν*NH_2_, *ν*C=O and *δ*N–C–C groups at 3400, 3350, 3180, 1690–1650 and 1620 cm^–1^. The absorption bands of aromatic rings, *ν*C–C_Ar_ 1650, 1580, 1400 cm^–1^ and *ν*C–H_Ar_ 3030, 3010 cm^–1^, were also observed. The molecular ion peak of cationic form with intensity of 100% was detected in the mass spectra of all investigated compounds.

**Figure 3:**
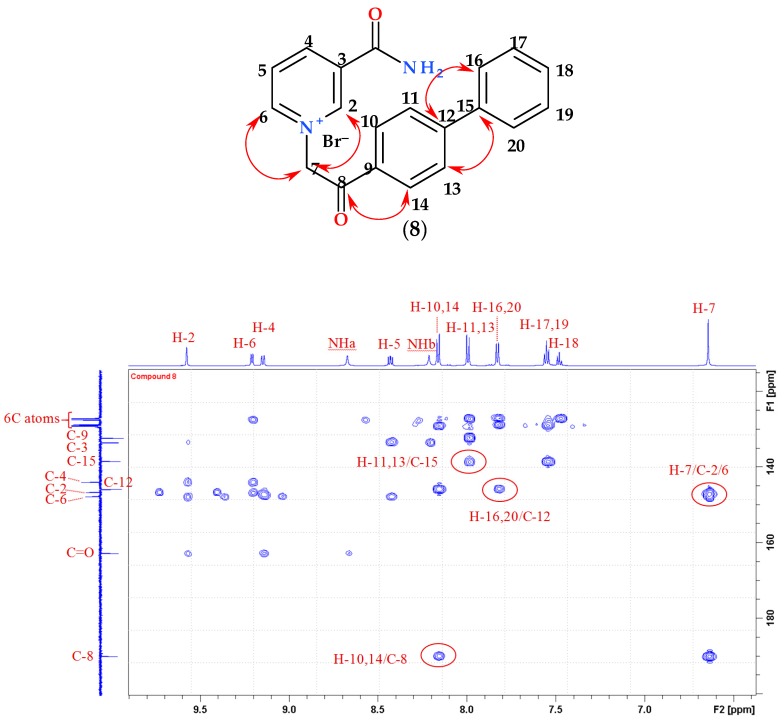
Structure and ^1^H-^13^C HMBC spectrum of (**8**) in DMSO-*d*_6_. Red arrows and circles mark the key correlation signals which proved the structure of obtained compound. The one-dimensional 600 MHz ^1^H-NMR spectrum is shown at the top edge and the 150 MHz ^13^C-NMR spectrum at the left-hand edge.

In the ^1^H-NMR spectra of all synthesized compounds (**1**–**10**) only one set of signals is present. The number, multiplicity and integrals of signals correspond to expected molecular structures. The enumeration scheme used for the assignment of the NMR spectra is presented in Figure 3. The aromatic area of ^1^H-NMR spectra contains four signals from pyridine moiety (from H-2 to H-6), two signals from the amide group (NH_2_) and two doublets, corresponding to the phenyl protons (H-10/14 and H-11/13, (**2**–**8**, **10**)). The aromatic area of the ^1^H-NMR spectrum of (**8**) also contains three signals from the substituted phenyl ring (H-16/20, H-17/19, H-18). The aromatic area of the ^1^H-NMR spectrum of (**9**) contains four different signals because of the –OCH_3_ group substituted in position C-14. In the ^1^H-NMR spectra only signals of H-4 and H-6 atoms were overlapping and they were distinguished by correlation signals in COSY spectra of (**2**) and (**8**). Signal of H-7 atom was observed at 6.55 ppm, and signals of –CH_3_ and –OCH_3_ group (**1**, **5**, **7**, **9**) were found from 2.44 to 4.42 ppm. In the ^13^C APT spectra, the most dshielded signals were of C-8 (189.6 ppm) and *C*ONH_2_ (162.7 ppm) nuclei. 

All aromatic ^13^C nuclei were found in the range 150–120 ppm, so each signal was assigned to its atom in the molecule by using C–H correlations over three bonds in HMBC spectra. The key correlation signals were found between atoms H-7 and C-2/6, H-10/14 and C-8, and in the spectra of (**8**) between H-16/20 and C-12 and H-11/13 and C-15 (Figure 3). These correlation signals unambiguously confirmed the structures of obtained compounds. The C-7 atom signal is at 66.3 ppm, while C-signals of methyl at 48.2 ppm (**1**) and at 21.2 ppm (**5**) and of methoxy groups at 55.8 ppm (**7**) and at 56.3 ppm (**9**). NMR spectra of synthesized compounds can be found in Supplementary files.

### 2.2. Antifungal Activity

Different concentration of compounds (**1**–**10**) were evaluated for their antifungal activities in vitro against four fungal pathogens (*Fusarium oxysporum*, *Fusarium culmorum*, *Macrophomina phaseolina, Sclerotinia sclerotiorum*), Table 3.

The newly synthesized compound (**3**) and (**4**) showed very good inhibitory activities against all fungal pathogens at concentrations of 10 and 100 µg/mL. More precisely, against *M. phaseolina* and *F. oxysporum*, great inhibitory activities at both concentrations were detected for compound (**3**), while against *F. culmorum*, (**4**) showed the highest inhibitory effects. The compound (**5**) showed the lowest activity at concentration of 10 µg/mL against *M. phaseolina*, while at concentration of 100 µg/mL the lowest activity was observed for compound (**8**). The compounds (**9**) and (**10**) indicate the lowest activity on mycelial growth of *F. culmorum*. All compounds tested here showed the great inhibitory activities against *S. sclerotiorum* at a concentration of 100 µg/mL.

Compounds (**1**), (**5**), (**7**), (**9**) and (**10**) also showed the strong inhibition of mycelium growth at a concentration of 10 µg/mL, although between them and compounds (**2**–**4**) and (**8**), there was no statistically significant difference. For *S. sclerotiorum* the same trend of mycelial growth inhibition is continued over the next 72 h and 96 h. Comparing fungicides and the prepared and tested compounds at both concentrations revealed no statistically significant difference between either, but the compounds will be further tested with intention of deepening their mechanism of action.

Literature data suggest that the mechanism of action of the fungicide derived from the strobilurin group proceeds through inhibition of mitochondrial respiration of fungi. The fungicides derived from the dithiocarbamates group are preventively and curatively fungicides, not systemic ones characterized by multiple-action.

The used fungicides are known as being highly efficient against *Fusarium* [25,26], *S. sclerotiorum* [27] and *M. phaseolina* fungal pathogens [28]. It was confirmed in other investigations that the pyridine carboxamide group of pesticides have a common target receptor, succinate dehydrogenase (SDH, EC 1.3.5.1.). They act as SDH inhibitors (SDHIs) and disrupt the mitochondrial tricarboxylic acid cycle and respiration chain [29].

## 3. Materials and Methods

### 3.1. General Experimental Procedures

All reactions were performed in a controllable single-mode microwave reactor by Microwave Synthesis Labstation Start S (Milestone, Shelton, CT, USA). The reactor is equipped with a magnetic stirrer as well as with temperature and power controls (220 V/50–60 Hz, 2.4 kW).

Solvents and reagents were purchased from Merck (Darmstadt, Germany) and used without further purification. To identify a compound by an investigation of its infrared spectrum Cary 630 FTIR, Agilent Technologies (Santa Clara, CA, USA) was used with DTGS (Deuterated triglycine sulfate) detector. NMR spectra were recorded on Bruker AV600 spectrometer (Bruker, Rheinstetten, Germany), operating at 600.135 MHz for the ^1^H nucleus and 150.903 MHz for the ^13^C nucleus. All data were recorded in DMSO-*d*_6_ at 298 K in 5 mm NMR tubes. Chemical shifts in ^1^H and ^13^C spectra (δ/ppm) were referenced to the methyl signal of tetramethylsilane (TMS). Proton spectra with spectral width of 12019 Hz and a digital resolution of 0.37 Hz per point were measured with 32 scans. ^13^C APT spectra with spectral width of 39,370 Hz and a digital resolution of 0.60 Hz per point, respectively, were collected with ≈300 scans. Assignments of the ^1^H- and ^13^C-NMR signals were performed using gradient-selected two-dimensional homo- and heteronuclear correlation experiments (^1^H-^1^H COSY, ^1^H-^13^C HMQC and ^1^H-^13^C HMBC). Melting points were determined with a Stuart melting point apparatus SMP^3^ (Mettler Toledo, Croatia).

### 3.2. Synthesis of 3-Carbamoyl-1-methylpyridinium Iodide (***1***)

*Conventional method (A):* Nicotinamide (0.244 g, 2 mmol) were dissolved in 10 mL of absolute ethanol and added to methyl iodide (0.187 mL, 3 mmol), which had also been previously dissolved in 10 mL of absolute ethanol. The reaction mixture was yellow after 17 h of reflux. Slow cooling of the reaction mixture promoted the formation of pure yellowish pearlescent needles crystals. They were washed with diethyl ether and recrystallized from methanol 

*MW synthesis in ethanol (B):* To nicotinamide (0.244 g, 2 mmol) dissolved in 10 mL of absolute ethanol, 0.187 mL of methyl iodide (3 mmol) was added. The reaction mixture was subjected to MW irradiation (20 min at 440 W) until the product was visible on TLC. Slow cooling of the reaction mixture promoted formation of pure yellowish pearlescent needles crystals. They were washed with diethyl ether and recrystallized from methanol.

*MW synthesis in acetone (B):* To nicotinamide (0.244 g, 2 mmol) dissolved in 5 mL of acetone, 0.187 mL of methyl iodide (3 mmol) was added. The reaction mixture was subjected to MW irradiation (20 min at 440 W) until the product was visible on TLC. Slow cooling of the reaction mixture promoted formation of pure yellowish pearlescent needles crystals. They were washed with diethyl ether and recrystallized from methanol.

*3-carbamoyl-1-methylpyridinium iodide* (**1**). Yield: conventional method, 411 mg, MW from ethanol 501 mg, MW from acetone 487 mg; m.p. 203–204 °C; IR (KBr) *ν*_max_ 3324.8, 3153.3, 3011.7, 2780.6, 1848.8, 1677.3, 1440.2, 1388.6, 1118.2, 775.3 cm^–1^; ^1^H-NMR (DMSO-*d*_6_, 600.135 MHz): δ 9.41 (1H, s, H-2), 9.12 (1H, d, *J* = 6.2 Hz, H-6), 8.91 (1H, td, *J* = 8.2, 1.59 Hz, H-4), 8.50 (1H, br s, N*Ha*), 8.26 (1H, dd, *J* = 8.1,6.1 Hz, H-5), 8.13 (1H, br s, N*Hb*), 4.42 (3H, s, CH_3_) ppm. ^13^C-NMR (DMSO-*d*_6_, 150.903 MHz): δ 162.8 (C, *C*ONH_2_) 147.1 (CH, C-6), 145.6 (CH, C-2), 142.8 (CH, C-4), 133.2 (C, C-3), 127.3 (CH, C-5), 48.2 (CH_3_, *C*H_3_) ppm; MS *m/z* 137 [M^+^] (100); anal. Calcd. For C_7_H_9_N_2_OI: C 31.84, H 3.44, I 48.06, N 10.61, O 6.06%. Found: C 31.40, H 3.27, N 10.48%.

### 3.3. Synthesis of Nicotinamide Derivatives with Substituted 2-Bromoacetophenones (***2***–***10***)

*Conventional method (A):* Nicotinamide (0.244 g, 2 mmol) and substituted 2-bromoacetophenone (2 mmol) were dissolved in 100 mL of acetone. The reaction mixture was mixed on a magnetic stirrer and heated for 1 h at 60 °C, and for another 24 h at room temperature. The crude product was filtered and washed with acetone. The reaction course was monitored by TLC chromatography with a mixture of chloroform: methanol (6:1). Recrystallization from appropriate solvent gave pure product.

*MW synthesis (B):* To a solution of nicotinamide (0.244 g, 2 mmol) in 10 mL of solvent (abs. ethanol or acetone), substituted 2-bromoacetophenones (2 mmol) were added and the reaction mixture was irradiated at 440 W for a 10 min. The crystals were removed from the solution by filtration, washed with diethyl ether and acetone and recrystallized from methanol.

*3-Carbamoyl-1-(2-(4-chlorophenyl)-2-oxoethyl)pyridin-1-ium bromide* (**2**). Yield: conventional method 227 mg, MW from abs. ethanol 480 mg, MW from acetone 462 mg; m.p. 252–254 °C, IR (KBr) *ν*_max_ 3486.8, 3198.1, 1689.8, 1650, 1590, 1394, 1341, 984, 902, 708.2, 620.2, 574 cm^–1^; ^1^H-NMR (DMSO-*d*_6_, 600.135 MHz): δ 9.51 (1H, s, H-2), 9.15 (1H, d, *J* = 6.1 Hz, H-6), 9.12 (1H, d, *J* = 8.2 Hz, H-4), 8.64 (1H, br s, N*Ha*), 8.41 (1H, dd, *J* = 8.2,6.1 Hz, H-5), 8.18 (1H, br s, N*Hb*), 8.08 (2H, d, *J* = 8.5 Hz, H-10, H-14), 7.76 (2H, d, *J* = 8.5 Hz, H-11, H-13), 6.56 (2H, s, H-7) ppm. ^13^C-NMR (DMSO-*d*_6_, 150.903 MHz): δ 189.7 (C, C-8), 162.7 (C, *C*ONH_2_), 147.7 (CH, C-6), 146.6 (CH, C-2), 144.0 (CH, C-4), 139.5 (C, C-12), 133.5 (C, C-3), 132.2 (C, C-9), 130.1 (CH, C-10, C-14), 129.2 (CH, C-11, C-13), 127.4 (CH, C-5), 66.3 (CH_2_, C-7) ppm; MS *m/z* 273.20 [M^+^](100); anal. Calcd. for C_14_H_12_BrClN_2_O_2_: C 47.29, H 3.40, Br 22.47, Cl 9.97, N 7.88, O 9.00. Found: C47.21, H 3.19, N 7.74%.

*1-(2-(4-Bromophenyl)-2-oxoethyl)-3-carbamoylpyridin-1-ium bromide* (**3**). Yield: conventional method 410 mg, MW from abs. ethanol 510 mg, MW from acetone 450 mg; m.p. 256–258 °C, IR (KBr) ν_max_ 3414.2, 3220.4, 2907.3, 1893.5, 1689.8, 1650, 1590, 1334.4, 1394, 1133.1, 984.0, 626.2, 566.6 cm^–1^; ^1^H-NMR (DMSO-*d*_6_, 600.135 MHz): δ 9.50 (1H, s, H-2), 9.13 (1H, dt, *J* = 6.2, 1.2 Hz, H-6), 9.11 (1H, dt, *J* = 8.2,1.2 Hz, H-4), 8.63 (1H, br s, N*Ha*), 8.40 (1H, dd, *J* = 8.2, 6.2 Hz, H-5), 8.18 (1H, br s, N*Hb*), 7.98 (2H, d, *J* = 8.8 Hz, H-10, H-14), 7.90 (2H, d, *J* = 8.6 Hz, H-11, H-13), 6.53 (2H, s, H-7) ppm. ^13^C-NMR (DMSO-*d*_6_, 150.903 MHz): δ 189.9 (C, C-8), 162.7 (C, *C*ONH_2_), 147.8 (CH, C-6), 146.7 (CH, C-2), 144.0 (CH, C-4), 133.5 (C, C-3), 132.6 (C, C-9), 132.2 (CH, C-11, C-13), 130.2 (CH, C-10, C-14), 128.8 (C, C-12), 127.5 (CH, C-5), 66.3 (CH_2_, C-7) ppm; MS *m/z* 321.10 [M^+^](100); anal. Calcd. For C_14_H_12_Br_2_N_2_O_2_: C 42.03, H 3.02, Br 39.95, N 7.00, O 8.00. Found: C 42.10, H 2.76, N 6.74%.

*3-Carbamoyl-1-(2-oxo-2-phenylethyl)pyridin-1-ium bromide* (**4**). Yield: conventional method 160 mg, MW from abs. ethanol 600 mg, MW from acetone 470 mg; m.p. 236.5–238 °C; IR (KBr) *ν*_max_ 3333.2, 3265.1, 3175.7, 2899.9, 2825.3, 2109.8, 1915.9, 1984.8, 1617.7, 1493.4, 1341.8, 1207.7, 888.9, 767.8 cm^–1^; ^1^H-NMR (DMSO-*d*_6_, 600.135 MHz): δ 9.57 (1H, s, H-2), 9.21 (1H, d, *J* = 6.0 Hz, H-6), 9.15 (1H, d, *J* = 8.3 Hz, H-4), 8.67 (1H, br s, N*Ha*), 8.42 (1H, dd, *J* = 8.0, 6.1 Hz, H-5), 8.21 (1H, br s, N*Hb*), 8.08 (2H, dd, *J* = 8.2, 1.1 Hz, H-10, H-14), 7.80 (1H, t, *J* = 7.4 Hz, H-12), 7.68 (2H, t, *J* = 8.2 Hz, H-11, H-13), 6.63 (2H, s, H-7) ppm. ^13^C-NMR (DMSO-*d*_6_, 150.903 MHz): δ 190.5 (C, C-8), 162.7 (C, *C*ONH_2_), 147.8 (CH, C-6), 146.6 (CH, C-2), 144.0 (CH, C-4), 134.7 (CH, C-12), 133.5 (C, C-9), 133.4 (C, C-3), 129.1 (CH, C-11, C-13), 128.2 (CH, C-10, C-14), 127.5 (CH, C-5), 66.3 (CH_2_, C-7) ppm; MS *m/z* 241 [M^+^](100); anal. Calcd. for C_14_H_13_N_2_O_2_Br: C 52.36, H 4.08, N 8.72. Found: C 52.07, H 3.78, N 8.67%.

*3-Carbamoyl-1-(2-oxo-2-(p-tolyl)ethyl)pyridin-1-ium bromide* (**5**). Yield: conventional method 120 mg, MW from abs. ethanol 590 mg, MW from acetone 510 mg; m.p. 248.3–248.8 °C; IR (KBr) ν_max_ 3578.2, 3332.2, 3235.3, 3168.2, 3056.4, 2937.1, 2117.1, 1878.6, 1684.8, 1610.2, 1463.7, 1401.5, 1334.4, 1215.1, 118.2, 991.5, 986, 909.5, 805.1 cm^−1^; ^1^H-NMR (DMSO-*d*_6_, 600.135 MHz): δ 9.55 (1H, s, H-2), 9.19 (1H, dt, *J* = 6.2, 0.9 Hz, H-6), 9.13 (1H, dt, *J* = 8.1, 1.3 Hz, H-4), 8.66 (1H, br s, N*Ha*), 8.41 (1H, dd, *J* = 8.1,6.2 Hz, H-5), 8.19 (1H, br s, N*Hb*), 7.97 (2H, d, *J* = 8.04 Hz,H-10, H-14), 7.47 (2H, d, *J* = 8.04 Hz, H-11, H-13), 6.58 (2H, s, H-7), 2.44 (3H, s, CH_3_) ppm. ^13^C-NMR (DMSO-*d*_6_, 150.903 MHz): δ 189.9 (C, C-8), 162.7 (C, *C*ONH_2_), 147.7 (CH, C-6), 146.5 (CH, C-2), 145.3 (C, C-12), 143.9 (CH, C-4), 133.4 (C, C-3), 130.9 (C, C-9), 129.6 (CH, C-11, C-13), 128.3 (CH, C-10, C-14), 127.4 (CH, C-5), 66.2 (CH_2_, C-7), 21.2 (CH_3_, C-15) ppm; MS *m/z* 255 [M^+^] (100); anal. Calcd. mass for C_15_H_15_BrN_2_O_2_:C 53.75, H 4.51, Br 23.84, N8.36, O 9.55. Found: C 52.62, H 4.49, N 8.35%.

*3-Carbamoyl-1-(2-(4-fluorophenyl)-2-oxoethyl)pyridin-1-ium bromide* (**6**). Yield: conventional method 250 mg, MW from abs. ethanol 480 mg, MW from acetone 309 mg; m.p. 246–249 °C; IR (KBr) ν_max_ 3518.5, 3332.2, 3235.3, 3175.7, 3078.8, 2937.1, 2117.1, 1900.9, 1684.8, 1595.3, 1401.5, 1326.9, 1230.0, 1103.3, 909.5, 752.9, 834.9, 685.8, 566.6 cm^–1^; ^1^H-NMR (DMSO-*d*_6_, 600.135 MHz): δ 9.52 (1H, s, H-2), 9.16 (1H, d, *J* = 6.1 Hz, H-6), 9.13(1H, dt, *J* = 8.2, 1.3 Hz, H-4), 8.64 (1H, br s, N*Ha*), 8.41 (1H, dd, *J* = 8.0, 6.0 Hz, H-5), 8.19 (1H, br s, N*Hb*), 8.16 (2H, m,H-10, H-14), 7.52 (2H, t, *J* = 8.8 Hz, H-11, H-13), 6.57 (2H, s, H-7) ppm. ^13^C-NMR (DMSO-*d*_6_, 150.903 MHz): δ 189.1 (C, C-8), 165.7 (C, *J*_C,F_ = 253 Hz, C-12), 162.7 (C, *C*ONH_2_), 147.7 (CH, C-6), 146.6 (CH, C-2), 144.0 (CH, C-4), 133.5 (C, C-3), 131.4 (CH, *J*_C,F_ = 10 Hz, C-10, C-14), 130.3 (C, *J*_C,F_ = 3 Hz, C-9), 127.4 (CH, C-5), 116.2 (CH, *J*_C,F_ = 22 Hz, C-11, C-13), 66.2 (CH_2_, C-7) ppm; MS *m/z* 259 [M^+^] (100); anal. Calcd for C_14_H_12_BrFN_2_O_2_:C 49.58, H3.57, N 8.26, O9.43. Found: C 48.48, H3.47, N 8.05%.

*3-Carbamoyl-1-(2-(4-methoxyphenyl)-2-oxoethyl)pyridin-1-ium bromide* (**7**). Yield: conventional method 80 mg, MW from abs. ethanol 630 mg, MW from acetone 530 mg; m.p. 238–240 °C; IR (KBr)ν_max_ 3257.7, 3123.5, 2952.1, 2947.7, 2117.1, 1900.9, 1677.3, 1602.8, 1401.5, 1244.9, 1125.7, 641.1, 605.1, 574.0, 395 cm^–1^; ^1^H-NMR (DMSO-*d*_6_, 600.135 MHz):δ 9.54 (1H, s, H-2), 9.19 (1H, dt, *J* = 6.1, 1.0 Hz,H-6), 9.13 (1H, dt, *J* = 8.1, 1.4 Hz, H-4), 8.65 (1H, br s, N*Ha*), 8.40 (1H, dd, *J* = 8.2,6.0 Hz, H-5), 8.19 (1H, br s, N*Hb*), 8.05 (2H, d, *J* = 8.8 Hz,H-10, H-14), 7.19 (2H, d, *J* = 8.8 Hz, H-11, H-13), 6.56 (2H, s, H-7), 3.91 (3H, s, H-15) ppm. ^13^C-NMR (DMSO-*d*_6_, 150.903 MHz): δ 188.7 (C, C-8), 163.3 (C, C-12), 162.8 (C, *C*ONH_2_), 147.8 (CH, C-6), 146.6 (CH, C-2), 143.9 (CH, C-4), 133.4 (C, C-3), 130.7 (CH, C-10, C-14), 127.4 (CH, C-5), 126.3 (C, C-9), 114.4 (CH, C-11, C-13), 66.7 (CH_2_, C-7), 55.8 (CH_3_, C-15) ppm; MS *m/z* 271 [M^+^] (100); anal. Calcd. for C_15_H_15_BrN_2_O_3_: C 51.30, H 4.31, Br 22.75, N 7.98, O 13.67. Found: C51.21, H 4.05, N 7.73%.

*1-(2-([1,1′-Biphenyl]-4-yl)-2-oxoethyl)-3-carbamoylpyridin-1-ium bromide* (**8**). Yield: conventional method 250 mg, MW from abs. ethanol 350 mg, MW from acetone 630 mg; m.p. 252.5–261 °C, IR (KBr) ν_max_ 3362.1, 3287.5, 3123.5, 2877.5, 2117.1, 1886, 1677.3, 1620, 1453.7, 1386.6, 1244.9, 1148, 998.9, 634.9, 775.3, 581.5 cm^–1^; ^1^H-NMR (DMSO-*d*_6_, 600.135 MHz): δ 9.58 (1H, s, H-2), 9.21 (1H, d, *J* = 6.1 Hz, H-6), 9.15 (1H, dt, *J* = 8.3, 1.4 Hz, H-4), 8.67 (1H, br s, N*Ha*), 8.43 (1H, dd, *J* = 8.1, 6.1 Hz, H-5), 8.21 (1H, br s, N*Hb*), 8.16 (2H, d, *J* = 8.7 Hz, H-10, H-14), 7.99 (2H, d, *J* = 8.5 Hz, H-11, H-13), 7.83 (2H, dd, *J* = 8.8, 1.4 Hz, H-16, H-20), 7.55 (2H, t, *J* = 7.2 Hz, H-17, H-19), 7.48 (1H, t, *J* = 7.2 Hz, H-18), 6.63 (2H, s, H-7) ppm. ^13^C-NMR (150.903 MHz, DMSO-*d*_6_, 25 °C): δ 190.0 (C, C-8), 162.7 (C, *C*ONH_2_), 147.7 (CH, C-6), 146.6 (C, C-2), 145.8 (1C, C-12), 144.0 (CH, C-4), 138.4 (C, C-15), 133.5 (C, C-3), 132.3 (C, C-9), 129.1 (CH, C-17, C-19), 129.0 (CH, C-10, C-14), 128.7 (CH, C-18), 127.4 (CH, C-5), 127.1 (CH, C-16, C-20), 127.0 (CH, C-11, C-13), 66.3 (CH_2_, C-7) ppm; MS *m/z*: 317 [M^+^] (100); anal. Calcd. for C_20_H_17_BrN_2_O_2_: C 60.47, H 4.31, Br 20.11, N 7.05, O 8.05. Found: C 60.52, H 4.14, N 6.68%.

*3-Carbamoyl-1-(2-(2-methoxyphenyl)-2-oxoethyl)pyridin-1-ium bromide* (**9**). Yield: conventional method 250 mg, MW from abs. ethanol 287 mg, MW from acetone 253 mg; m.p. 245–247 °C; IR (KBr) ν_max_ 3339.7, 3235.3, 3135.3, 2952.1, 2117.1, 1886.0, 1684.8, 1595.3, 1461.1, 1386.6, 1289.7, 1207.7, 909.5, 760.4, 678.4, 588.6 cm^–1^; ^1^H-NMR (DMSO-*d*_6_, 600.135 MHz): δ 9.54 (1H, s, H-2), 9.17 (1H, d, *J* = 6.0 Hz, H-6), 9.10 (1H, dt, *J* = 8.3, 1.2 Hz, H-4), 8.63 (1H, br s, N*Ha*), 8.38 (1H, dd, *J* = 8.0, 6.2 Hz, H-5), 8.17 (1H, br s, N*Hb*), 7.89 (1H, dd, *J* = 7.8, 1.8 Hz, H-10), 7.75 (1H, ddd, *J* = 7.3, 1.8 Hz, H-12), 7.36 (1H, d, *J* = 8.4 Hz, H-13), 7.16 (1H, m, H-11), 6.33 (2H, s, H-7), 4.05 (3H, s, OC*H*_3_) ppm. ^13^C-NMR (DMSO-*d*_6_, 150.903 MHz): δ 189.8 (C, C-8), 162.7 (C, *C*ONH_2_), 160.0 (C, C-14), 147.7 (CH, C-6), 146.4 (CH, C-2), 143.8 (CH, C-4), 136.3 (CH, C-12), 133.3 (C, C-3), 130.3 (CH, C-10), 127.2 (CH, C-5), 122.8 (C, C-9), 120.9 (CH, C-11), 113.1 (CH, C-13), 70.0 (CH_2_, C-7), 56.3 (CH_3_, C-15) ppm; MS *m/z* 271 [M^+^] (100); anal. Calcd. for C_15_H_15_BrN_2_O_3_: C 51.30, H 4.31, Br 22.75, N 7.98, O 13.67. Found: C 51.50, H 4.39, N 7.77%.

*3-Carbamoyl-1-[2-(4-nitrophenyl)-2-oxoethyl]pyridinium bromide* (**10**). Yield: conventional method 252 mg, MW from abs. ethanol 277 mg, MW from acetone 271 mg; m.p. 262.0–262.5 °C; IR (KBr)ν_max_ 1707, 1525–1494, 1950, 1558, 1373 cm^–1^; ^1^H-NMR (DMSO-*d*_6_, 600.135 MHz): δ 9.57 (1H, s, H-2), 9.21 (1H, d, *J*= 6.0 Hz, H-6), 9.17 (1H, d, *J*= 8.0 Hz, H-4), 8.69 (1H, br s, N*Ha*), 8.48 (2H, d, *J* = 8.80 Hz, H-11, H-13), 8.44 (1H, dd, *J*= 8.0, 6.0 Hz, H-5), 8.32 (2H, d, *J* = 8.8 Hz, H-10, H-14), 8.22 (1H,br s, N*Hb*), 6.68 (2H, s, H-7) ppm. ^13^C-NMR (DMSO-*d*_6_, 150.903 MHz): δ 189.9 (C, C-8), 162.8 (C, *C*ONH_2_), 150.6 (C, C-12), 147.8 (CH, C-2), 146.8 (CH, C-6), 144.1 (CH, C-4), 138.2 (C, C-9), 133.5 (C, C-3), 129.7 (CH, C-10, C-14), 127.6 (CH, C-5), 124.1 (CH, C-11, C-13), 66.7 (CH_2_, C-7) ppm; MS *m/z* 367 [M^+^] (100); anal. Calcd. for C_13_H_11_N_2_O_3_Br: C 48.32, H 3.43, N 8.67. Found: C 48.74, H 3.36, N 8.77%.

### 3.4. Antifungal Assay

Antifungal assay was performed on four cultures of phytopathogenic fungi (*Fusarium oxysporum*, *Fusarium culmorum*, *Macrophomina phaseolina* and *Sclerotinia sclerotiorum*) provided from the culture collections of the Chair for phytopathology, Faculty of Agrobiotechnical Sciences, Josip Juraj Strossmayer University of Osijek, Croatia. The fungicidal activity of ten synthetic compounds at concentrations of 10 µg/mL and 100 µg/mL were tested.

For antifungal assays sterilized Petri dish (9 cm in diameter) were used. Each Petri dish was filled with 10 mL of mix of PDA and one of synthetic compounds of specific concentrations. Agar plugs (4 mm diameter) of one week old culture were picked up with a sterilized needle and placed in the center of the Petri dishes. For antifungal assays was used according to the method proposed by Wu et al. [8]. Petri dishes were stored in the chamber at 22 ± 1 °C, with 12 h light/12 h dark regime and 70% RH. The fungi colony diameter of each culture was measured 48 h after inoculation.

Each treatment was performed in four replicates. As a control 1% DMSO was used. Commercially agricultural fungicide was used as positive control. The fungicide Amistar Extra with active ingredient from the strobilurin group was used for *F. culmorum*, *F. oxysporum* and *S.sclerotiorum*, while the fungicide Star 80 WP with active ingredient from the dithiocarbamates group was used for *M. phaseolina*. The fungicides were mixed with PDA in the recommended concentration.

Statistical analysis of data was performed using factorial analysis of variance ANOVA by grouping the data depending on coating and concentration applied. Fisher’s LSD test was applied to estimate the statistical significance of differences between different synthetic compounds and concentrations using SAS 9.2 Statistical Package (SAS Institute Inc., Cary, NC, USA) [30]. Mean values were considered significantly different when *p* ≤ 0.05.

## 4. Conclusions

The quaternization reactions of nicotinamide with different electrophiles—methyl iodide and 2-bromoacetophenones derivatives were carried out by conventional synthesis and by microwave irradiation synthesis, in absolute ethanol and acetone. By using microwave dielectric heating we obtained significantly improved yields, up to 8 times greater, and reduced reaction times down to 10–20 min. The synthesized compounds have been screened for antifungal activities against *Fusarium oxysporum*, *Fusarium culmorum*, *Macrophomina phaseolina* and *Sclerotinia sclerotiorum* at concentrations of 10 µg/mL and 100 µg/mL. Six compounds showed the strong inhibition of mycelium growth at concentration of 10 µg/mL, while all tested compounds showed the great inhibitory activities against *S. sclerotiorum* at a concentration of 100 µg/mL. Such investigations offer an alternative for more environmentally acceptable fungicides.

## Figures and Tables

**Table 1:** Screening of reaction times in the quaternization of nicotinamide and 2-bromoacetophenone (**4**) at 440 W.

Time (min)	2	4	6	8	10	12	14	16
Yield (%)	22	28	55	71	93	85	89	90

**Table 2:** Experimental times and yields of products (**1**–**10**) obtained by conventional method and by microwave-assisted method, in ethanol and acetone.

Compound	Conventional Method (A)			Microwave-Assisted Method (B)		
	Solvent	Time (h)	Yield (%)	Time (min)	EtOH	Acetone
					Yield (%)	Yield (%)
**(1)**	EtOH	17 (rt)	78	20	95	92
**(2)**	Acetone	1 (60 °C) + 24 (rt)	32	10	67	65
**(3)**			51		64	56
**(4)**			25		93	73
**(5)**			18		88	76
**(6)**			37		71	46
**(7)**			11		90	75
**(8)**			31		44	80
**(9)**			36		41	36
**(10)**			39		43	42

**Table 3:** The effect of different compounds on mycelial growth (mm) at 10 µg/mL and 100 µg/mL concentrations.

Mycelial Growth (mm)								
**Compounds**	*M. phaseolina*		*F. culmorum*		*F. oxysporum*		*S. sclerotiorum*	
	10 µg/mL	100 µg/mL	10 µg/mL	100 µg/mL	10 µg/mL	100 µg/mL	10 µg/mL	100 µg/mL
(**1**)	21.5 ± 5.8	16.75 ± 3.2	25.25 ± 3	19.25 ± 0.9	15 ± 1.4	12.75 ± 1.2	4 ± 0	4 ± 0
(**2**)	18.75 ± 4.6	18.75 ± 4.3	23.75 ± 1.5	20.25 ± 1.7	15 ± 2.8	14 ± 1.6	4.5 ± 1	4 ± 0
(**3**)	16.25 ± 4.5	15 ± 4.8	24 ± 0	19.5 ± 0.6	15 ± 1.4	12.25 ± 0.9	4.25 ± 0.5	4 ± 0
(**4**)	20.5 ± 5.2	1.9 ± 4.5	22.5 ± 1.9	18.5 ± 0.6	15.5 ± 0.6	13.5 ± 2.4	4.75 ± 0.9	4 ± 0
(**5**)	23.75 ± 3.9	23 ± 3.4	25.75 ± 1.5	26 ± 1.8	16.5 ± 1	12.5 ± 1.3	4 ± 0	4 ± 0
(**6**)	21 ± 6.2	21 ± 5.2	26.5 ± 2.6	26 ± 2.4	15 ± 1.6	12.5 ± 0.6	4 ± 0	4 ± 0
(**7**)	20.5 ± 4.2	20 ± 8.1	26.5 ± 3.1	25 ± 2.4	13.75 ± 1.2	12.25 ± 0.9	4 ± 0	4 ± 0
(**8**)	16.5 ± 6.4	24.2 ± 3.9	26.5 ± 2.2	31.75 ± 1.2	14.5 ± 1	16.25 ± 0.9	4.25 ± 0.5	4 ± 0
(**9**)	20.5 ± 5.8	22 ± 2.9	29.5 ± 1.3	29.75 ± 0.9	16 ± 0.8	14.25 ± 0.9	4.5 ± 1	4 ± 0
(**10**)	22.25 ± 2.2	19 ± 3.6	29 ± 1.1	31.75 ± 0.5	16.25 ± 0.5	15 ± 0.8	4 ± 0	4 ± 0
**Strobilurin ^a^**			4 ± 0	4 ± 0	4 ± 0	4 ± 0	4 ± 0	4 ± 0
**Dithio-carbamates ^a^**	4 ± 0	4 ± 0						
**LSD test**	6.91	6.34	2.79	2.06	1.91	1.74	0.80	0

Mean ± SD determinated from Fhiser’s test, when these means were compared with controls, mean values were considered significantly different when *p* ≤ 0.05 [30]. ^a^ The commercial fungicide strobilurin and dithiocarbamates were used for comparison.

## Supplementary Materials

The following are available online, copies of ^1^H- and ^13^C-NMR spectra, copies of ^1^H-^1^H COSY NMR spectra of compounds (**2**) and (**8**), copy of ^1^H-13C HMQC NMR spectrum of compound (**8**), copies of 1H-13C HMBC NMR spectra of compounds (**2**), (**3**) and (**9**).
