# Supplementary material for: An Improved Method for the Quaternization of Nicotinamide and Antifungal Activities of Its Derivatives
Source: Molecules. 2019 Mar 13;24(6):1001. doi: 10.3390/molecules24061001 (PMC6470469; doi:10.3390/molecules24061001)
Supplement: Supplementary file 1 [file molecules-24-01001-s001.pdf]

Supporting Information

An Improved, Efficient Method for the Quaternization of Nicotinamide and Antifungal Activities of Its Derivatives

Tamara Siber[1], Valentina Bušić[2], Dora Zobundžija[2], Sunčica Roca[3], Dražen Vikić-Topić[3], Karolina Vrandečić[1] andDajana Gašo-Sokač[2*]

[1] Faculty of Agrobiotechnical Sciences, Josip Juraj Strossmayer University of Osijek, Vladimira Preloga 1, HR–31000 Osijek, Croatia

[2] Faculty of Food Technology, Josip Juraj Strossmayer University of Osijek, Kuhačeva 20, HR–31000 Osijek, Croatia

[3] NMR Centre, Ruđer Bošković Institute, Bijenička cesta 54, HR–10000 Zagreb, Croatia

Department of Natural and Health Sciences, Juraj Dobrila University of Pula, Zagrebačka 30, HR–52100 Pula, Croatia

*Correspondence: dajana.gaso@ptfos.hr; Tel.: +385-31-224327.

Contents

I) Copies of ^{1}H- and ^{13}C-NMR spectra 1

II) Copies of ^{1}H-^{1}H COSY NMR spectra of compounds **(2)** and **(8)** 11

III) Copy of ^{1}H-^{13}C HMQC NMR spectrum of compound **(8)** 12

IV) Copies of ^{1}H-^{13}C HMBC NMR spectra of compounds **(2), (3)** and **(9)** 13

I) Copies of ^{1}H- and ^{13}C-NMR spectra

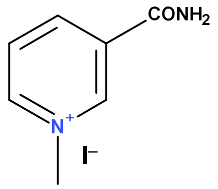

(1)

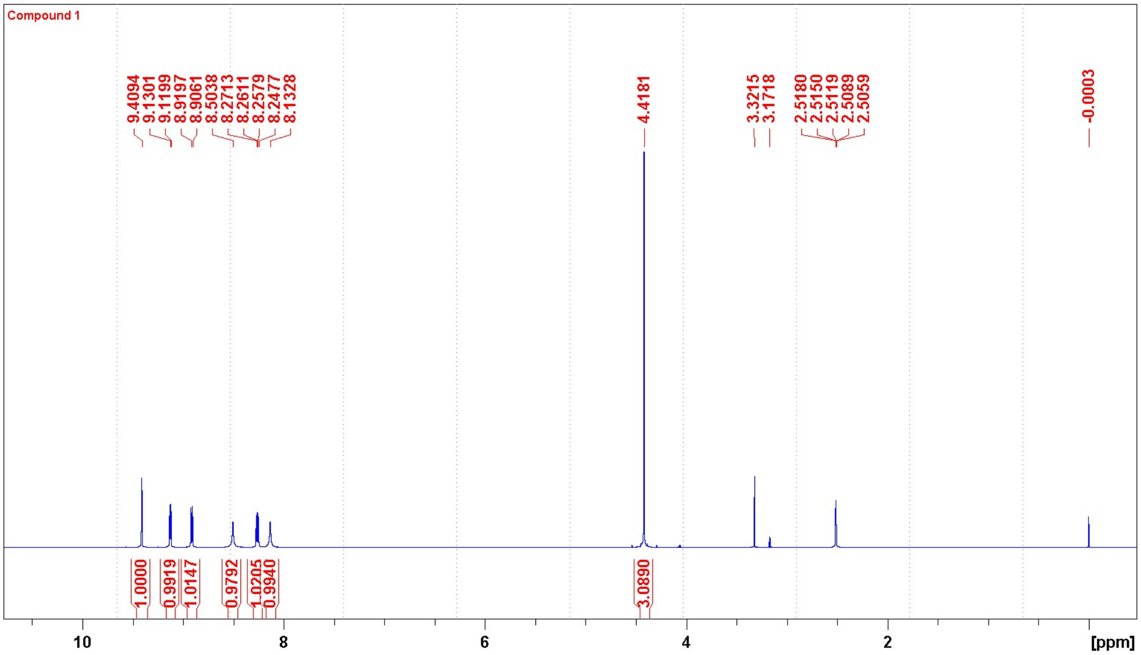

Figure S1. ^{1}H NMR (600 MHz, DMSO-d_6) spectrum of the compound **(1)**.

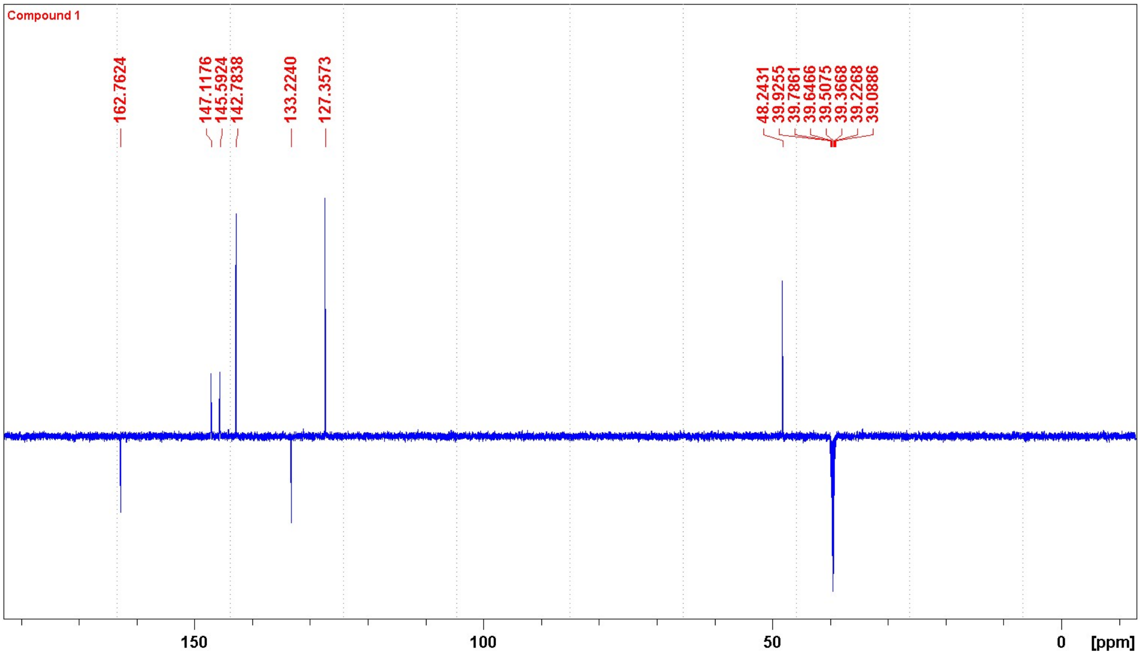

Figure S2. ^{13}C APT NMR (150 MHz, DMSO-d_6) spectrum of the compound **(1)**.

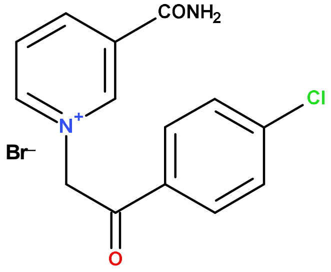

(2)

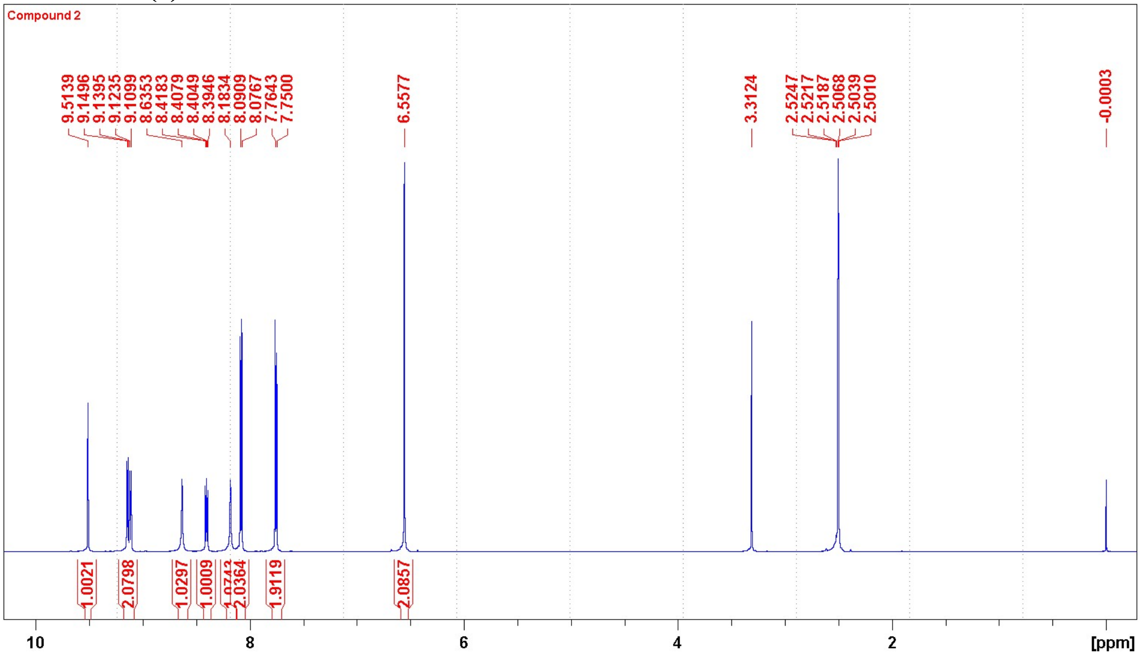

Figure S3. ^{1}H NMR (600 MHz, DMSO-d_6) spectrum of the compound **(2)**.

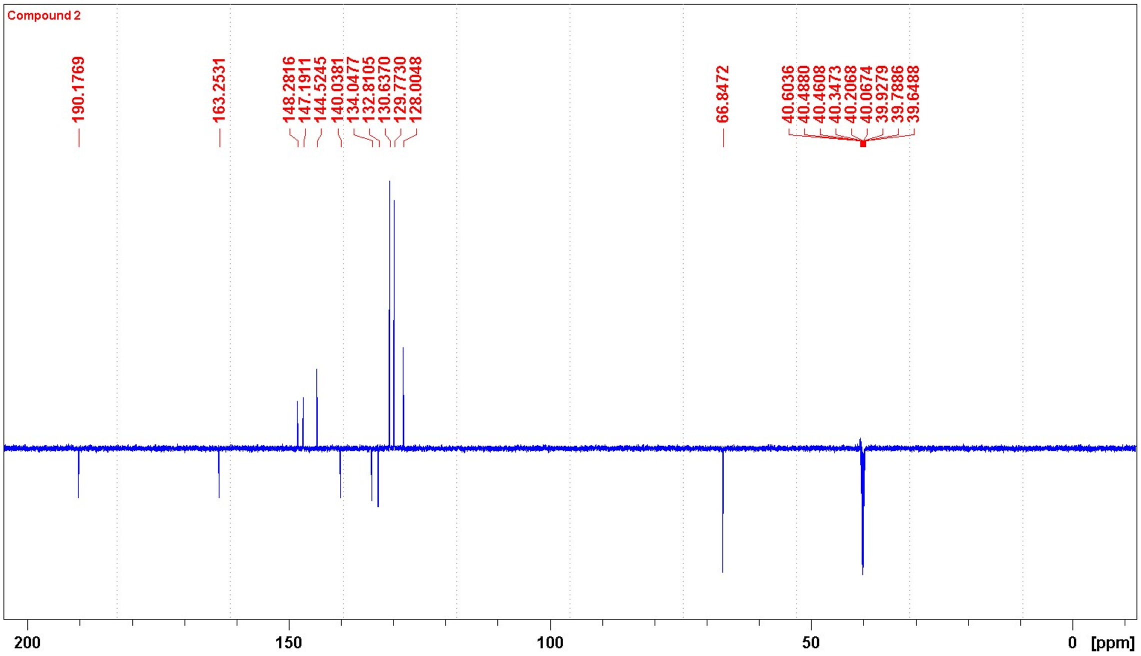

Figure S4. ^{13}C APT NMR (150 MHz, DMSO-d_6) spectrum of the compound **(2)**.

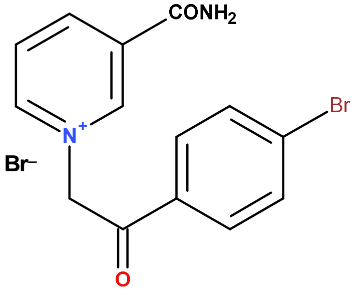

(3)

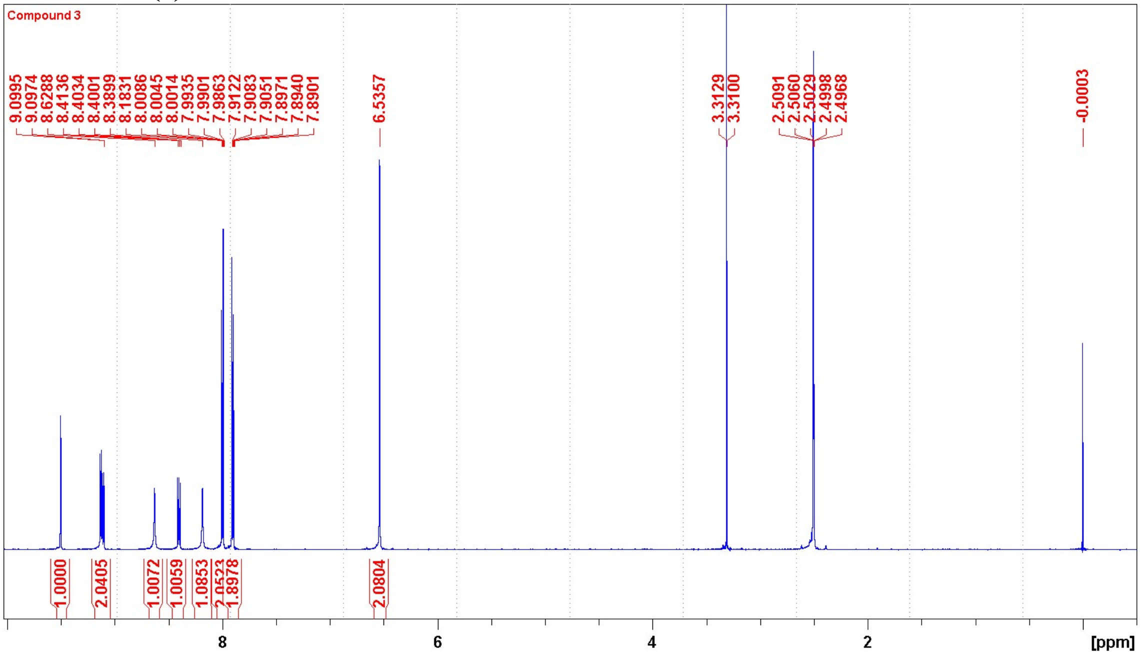

Figure S5. ^{1}H NMR (600 MHz, DMSO-d_6) spectrum of the compound **(3)**.

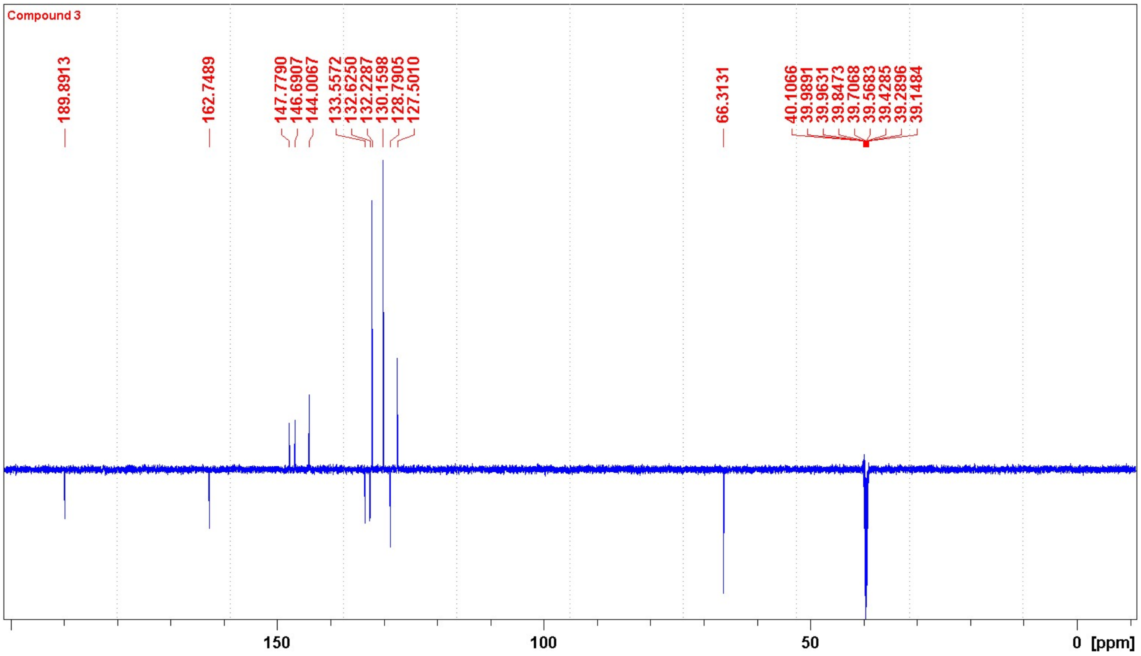

Figure S6. ^{13}C APT NMR (150 MHz, DMSO-d_6) spectrum of the compound **(3)**.

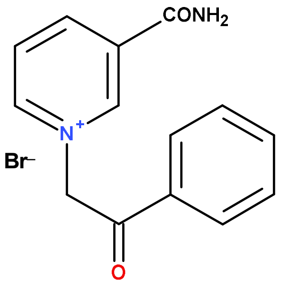

(4)

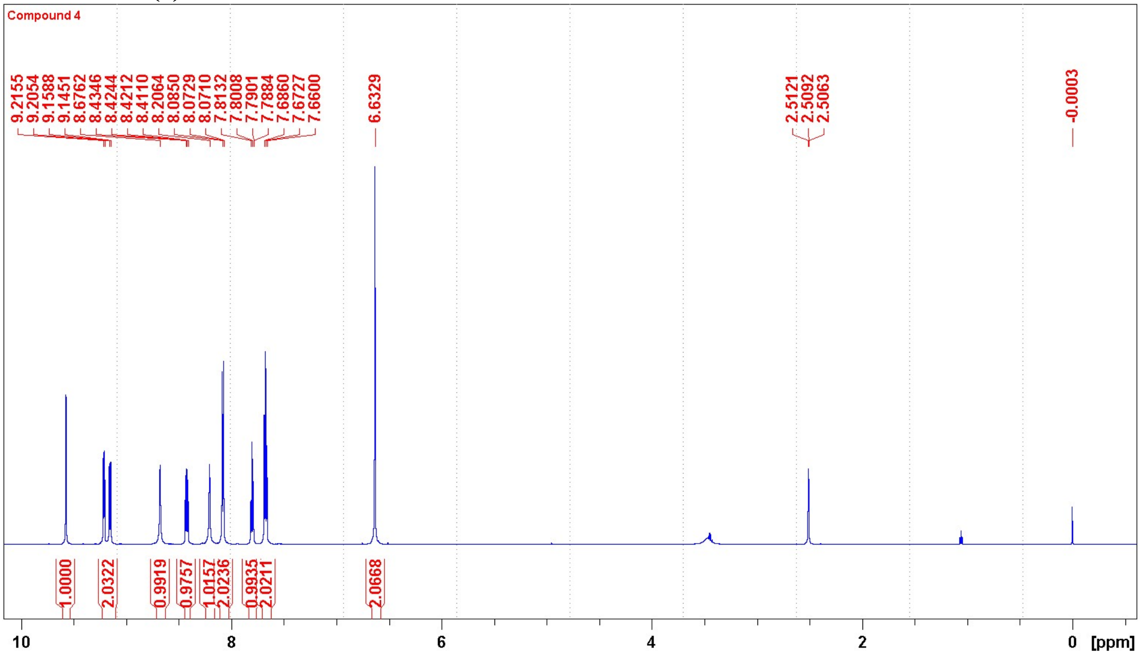

Figure S7. ^{1}H NMR (600 MHz, DMSO-d_6) spectrum of the compound **(4)**.

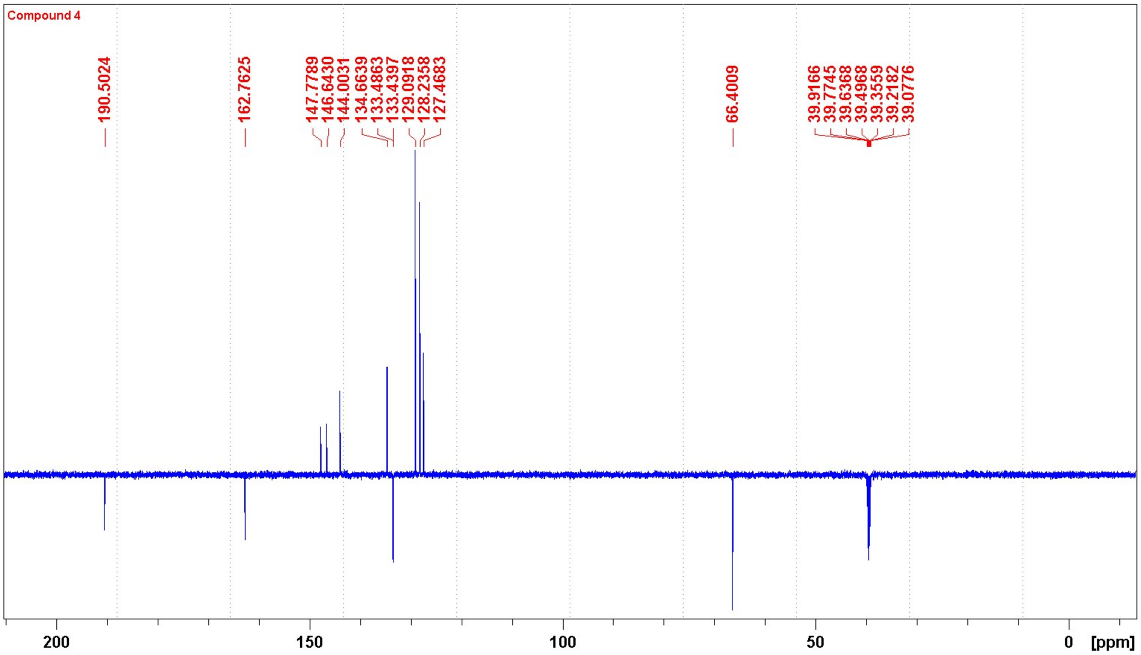

Figure S8. ^{13}C APT NMR (150 MHz, DMSO-d_6) spectrum of the compound **(4)**.

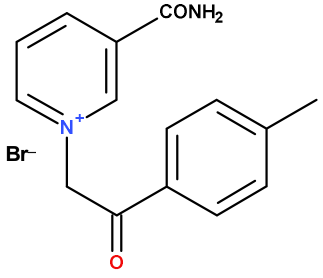

(5)

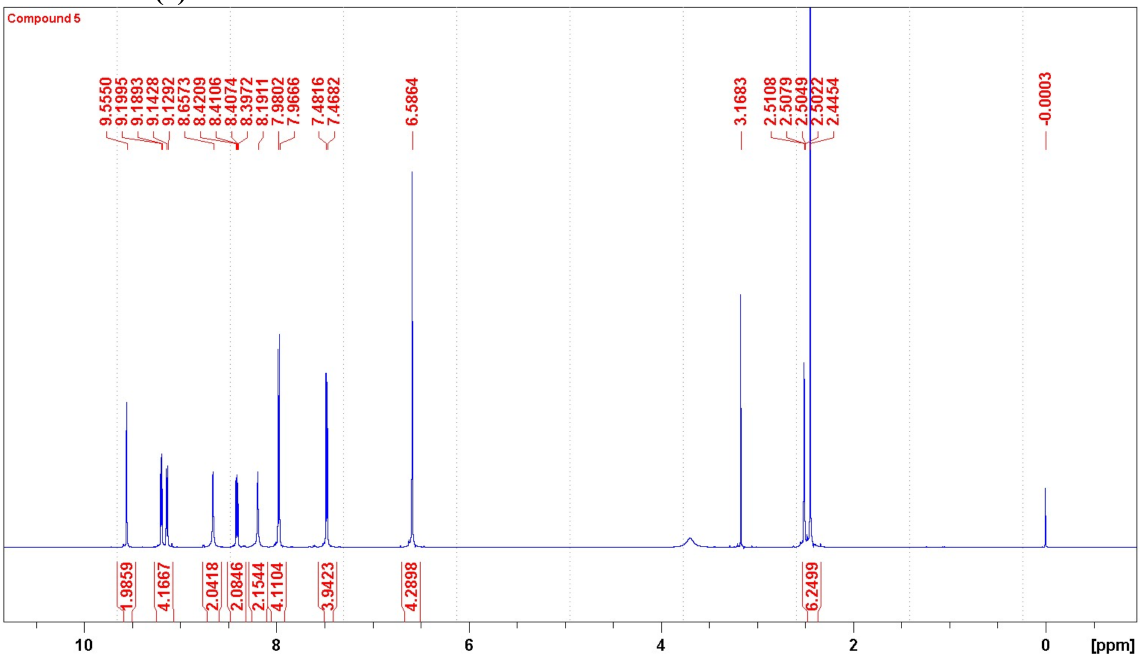

Figure S9. ^{1}H NMR (600 MHz, DMSO-d_6) spectrum of the compound **(5)**.

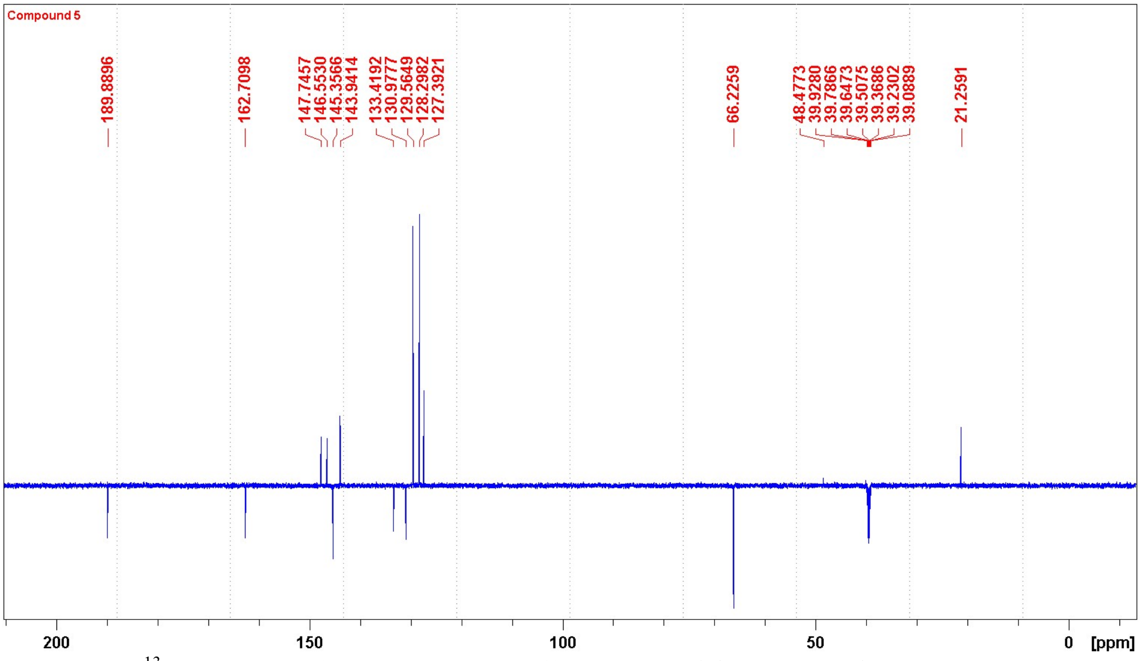

Figure S10. ^{13}C APT NMR (150 MHz, DMSO-d_6) spectrum of the compound **(5)**.

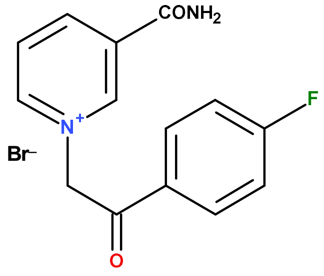

(6)

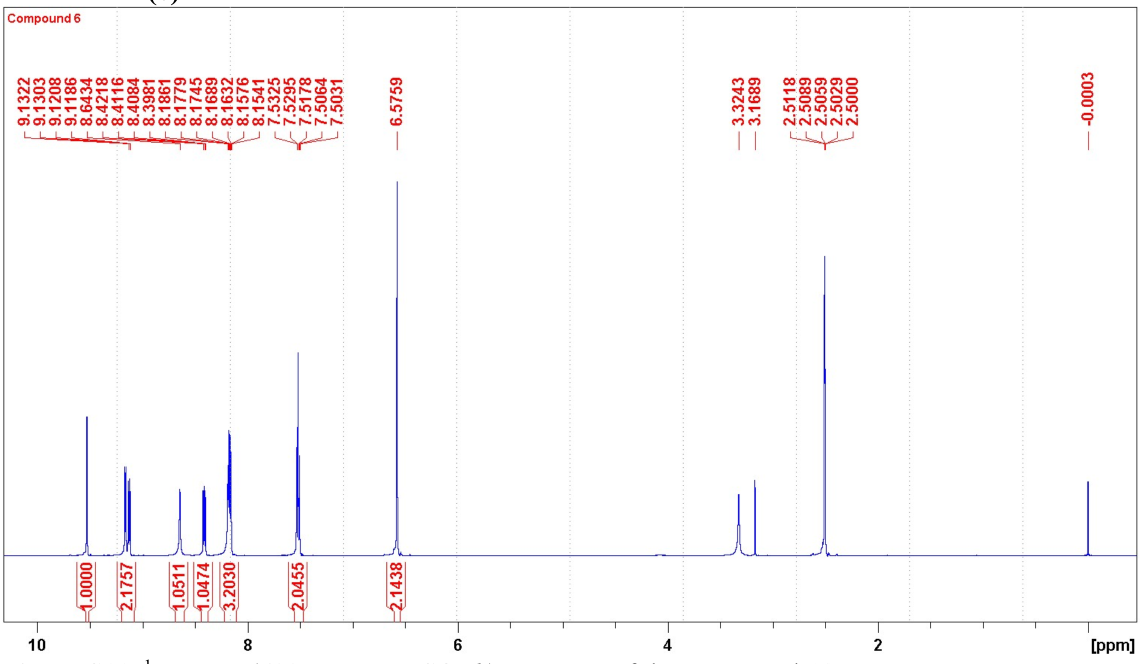

Figure S11. ^{1}H NMR (600 MHz, DMSO-d_6) spectrum of the compound **(6)**.

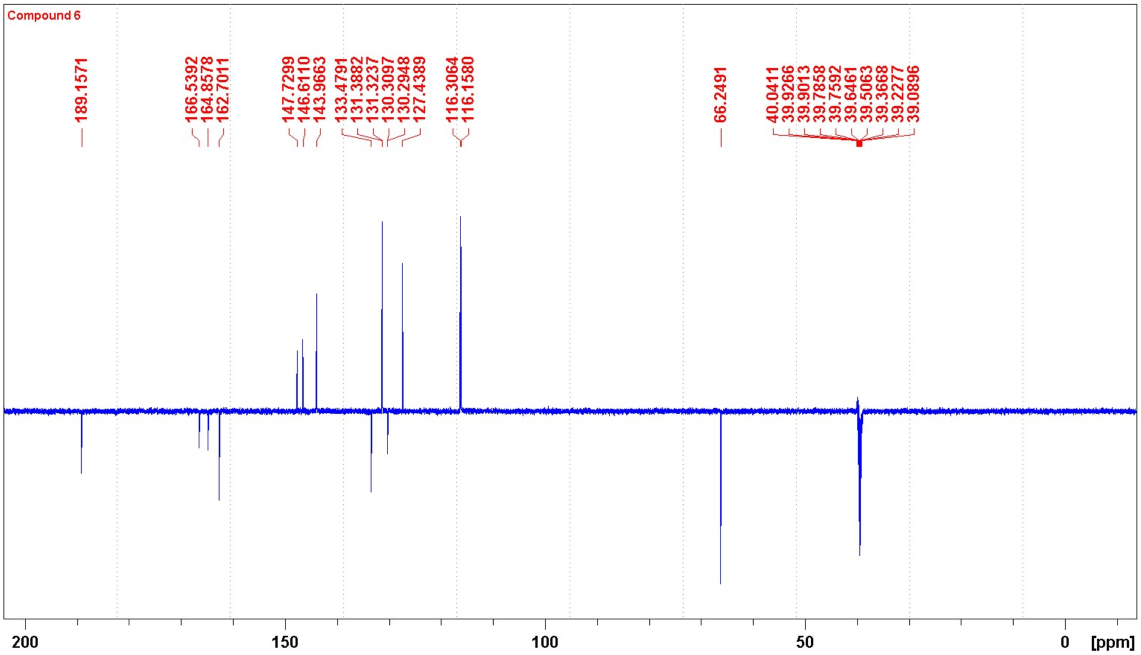

Figure S12. ^{13}C APT NMR (150 MHz, DMSO-d_6) spectrum of the compound **(6)**.

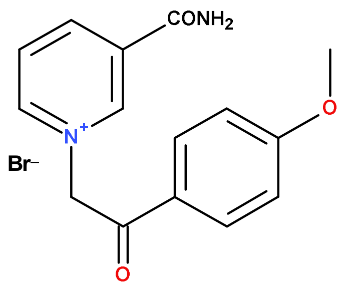

(7)

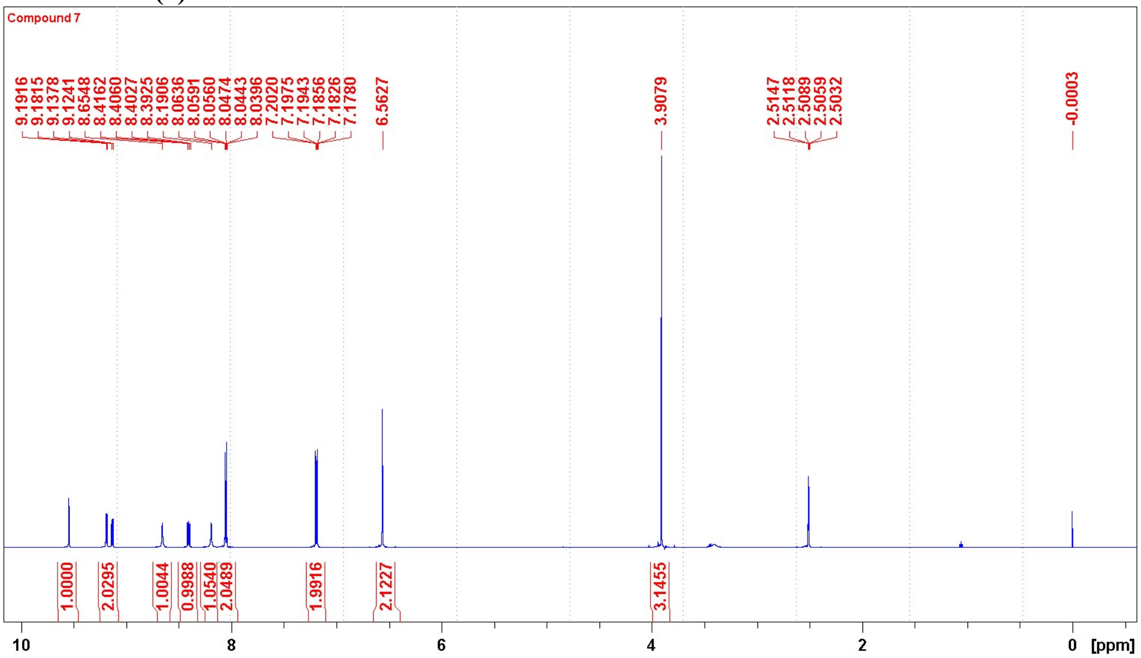

Figure S13. ^{1}H NMR (600 MHz, DMSO-d_6) spectrum of the compound **(7)**.

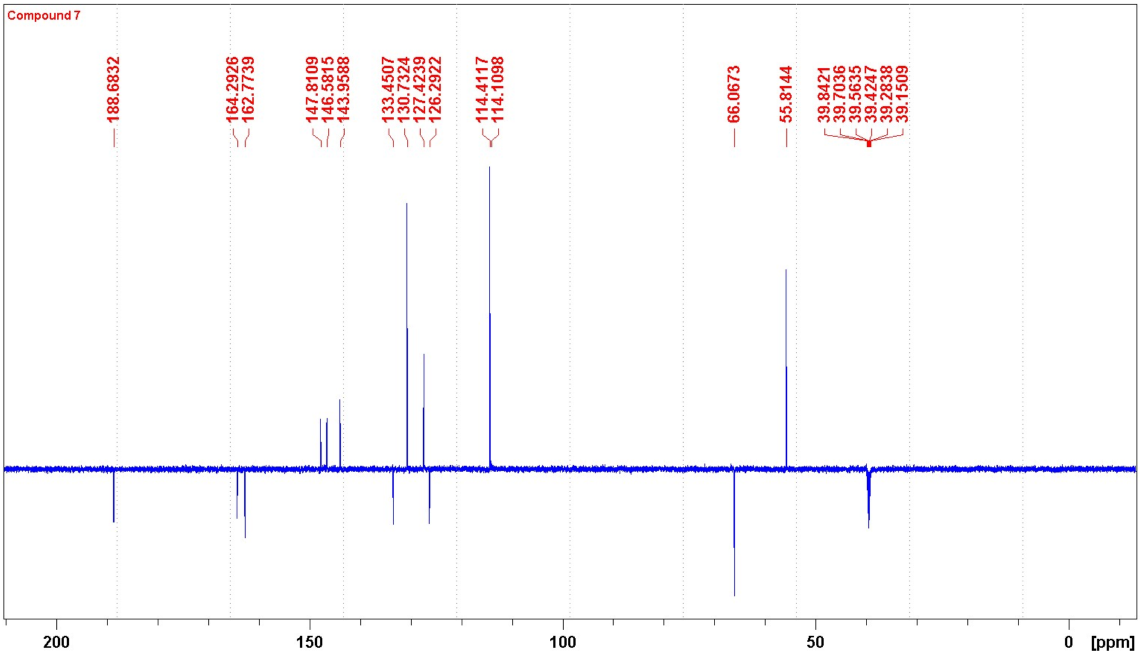

Figure S14. ^{13}C APT NMR (150 MHz, DMSO-d_6) spectrum of the compound **(7)**.

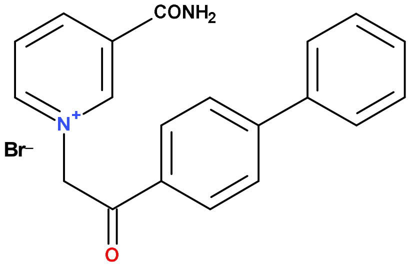

(8)

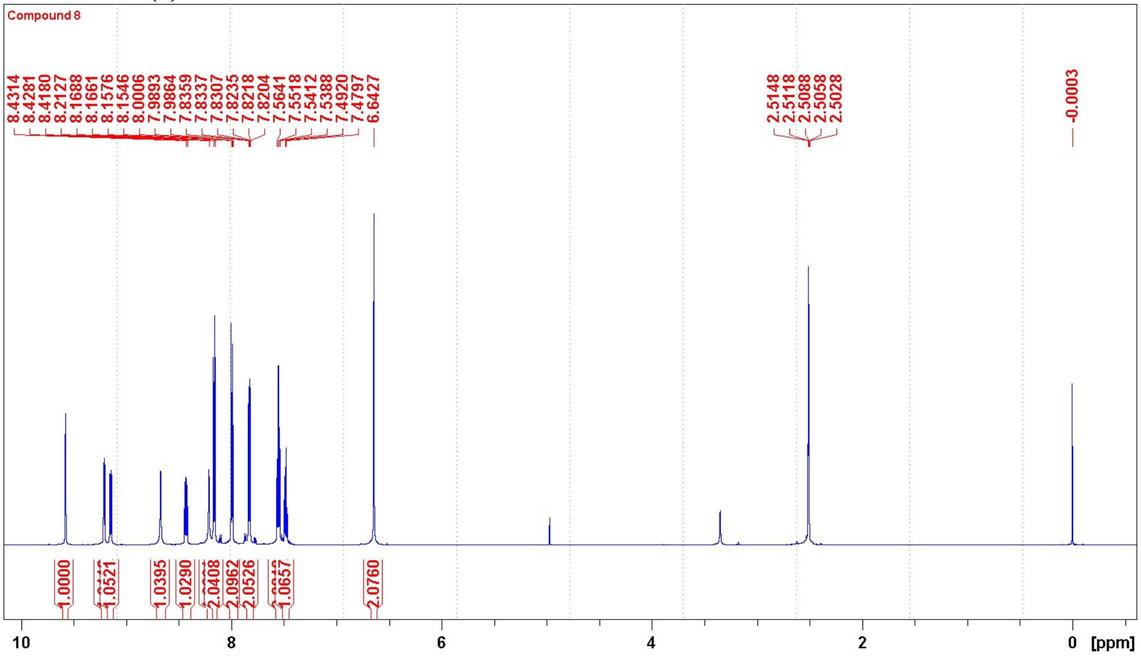

Figure S15. ^{1}H NMR (600 MHz, DMSO-d_6) spectrum of the compound **(8)**.

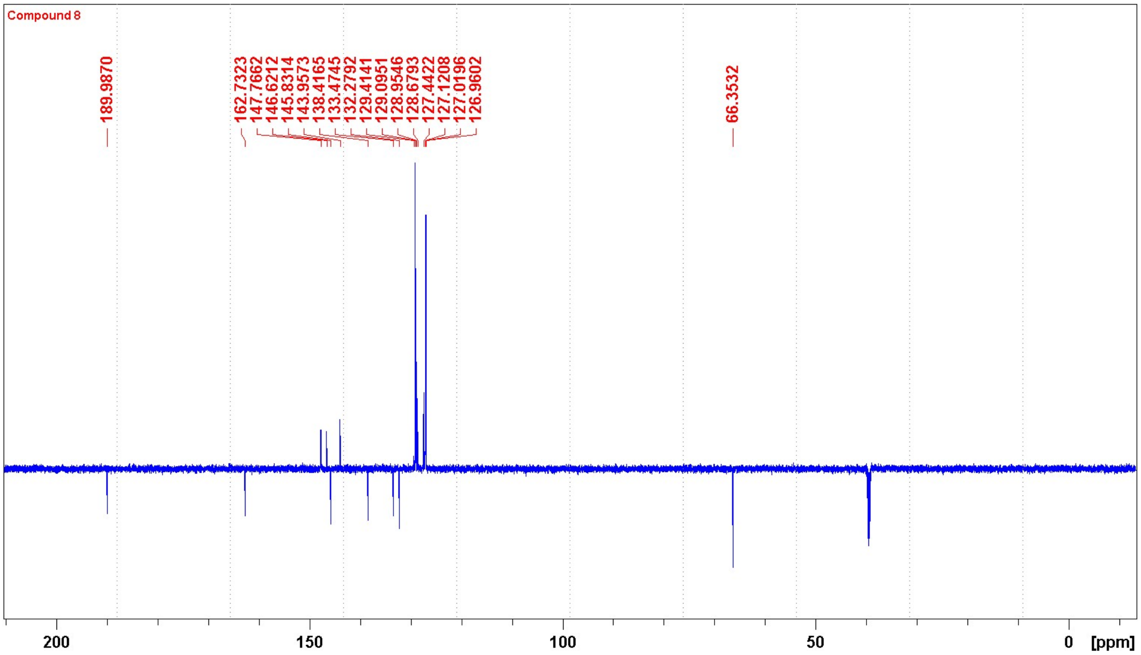

Figure S16. ^{13}C APT NMR (150 MHz, DMSO-d_6) spectrum of the compound **(8)**.

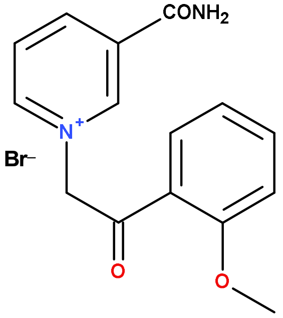

(9)

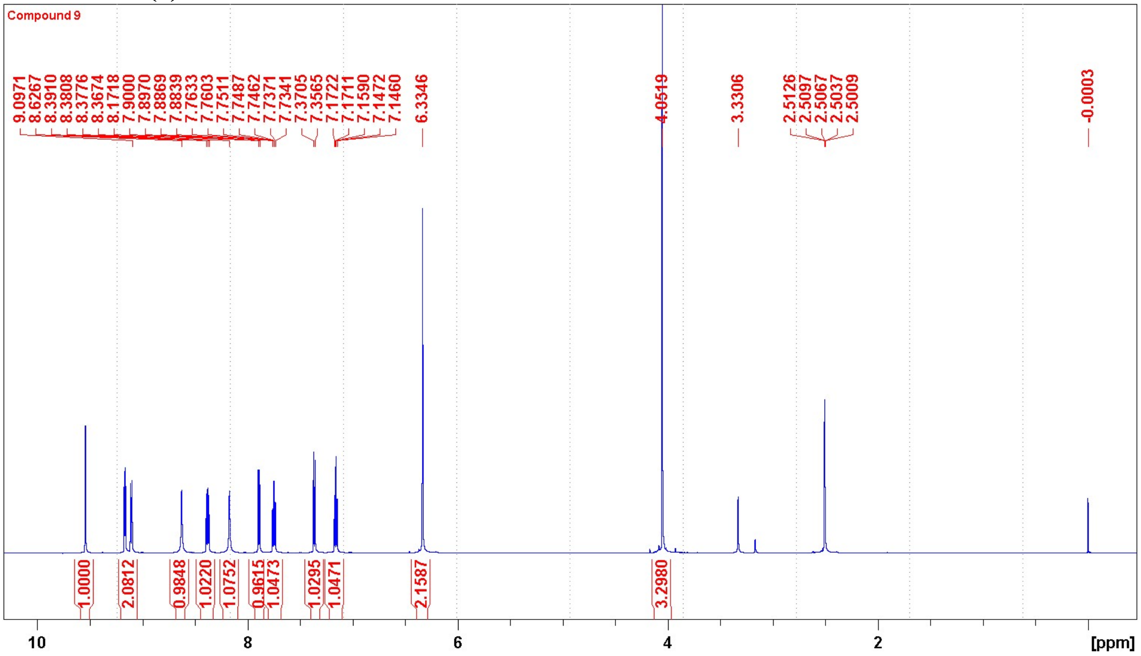

Figure S17. ^{1}H NMR (600 MHz, DMSO-d_6) spectrum of the compound **(9)**.

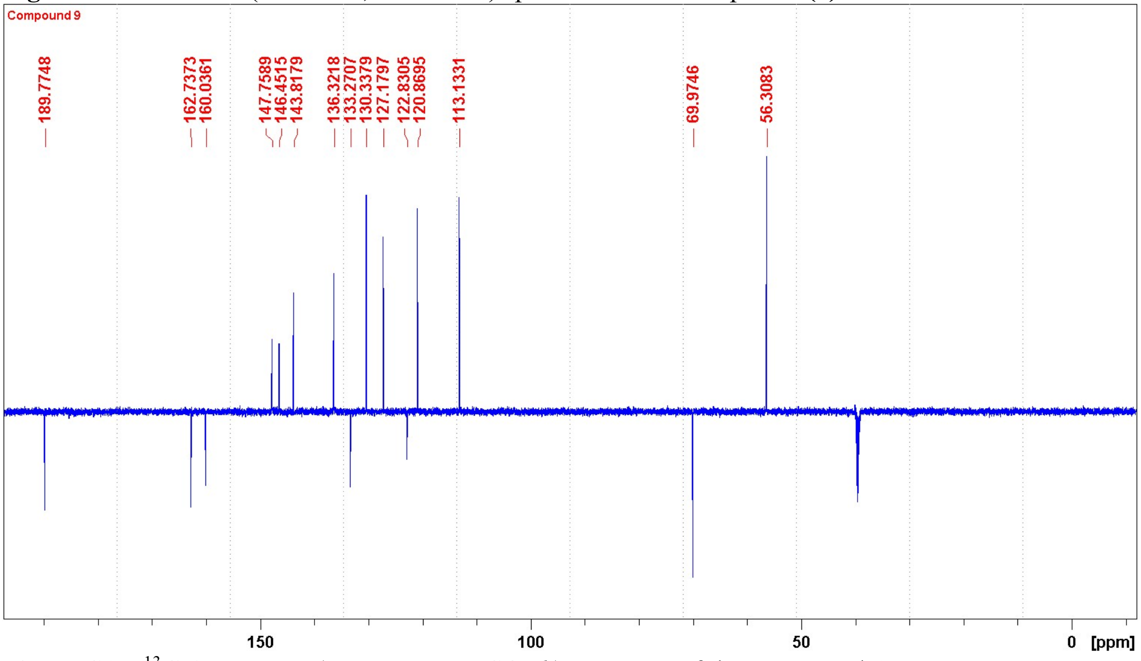

Figure S18. ^{13}C APT NMR (150 MHz, DMSO-d_6) spectrum of the compound **(9)**.

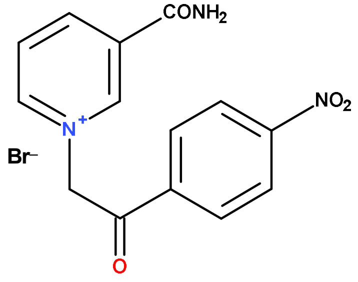

(10)

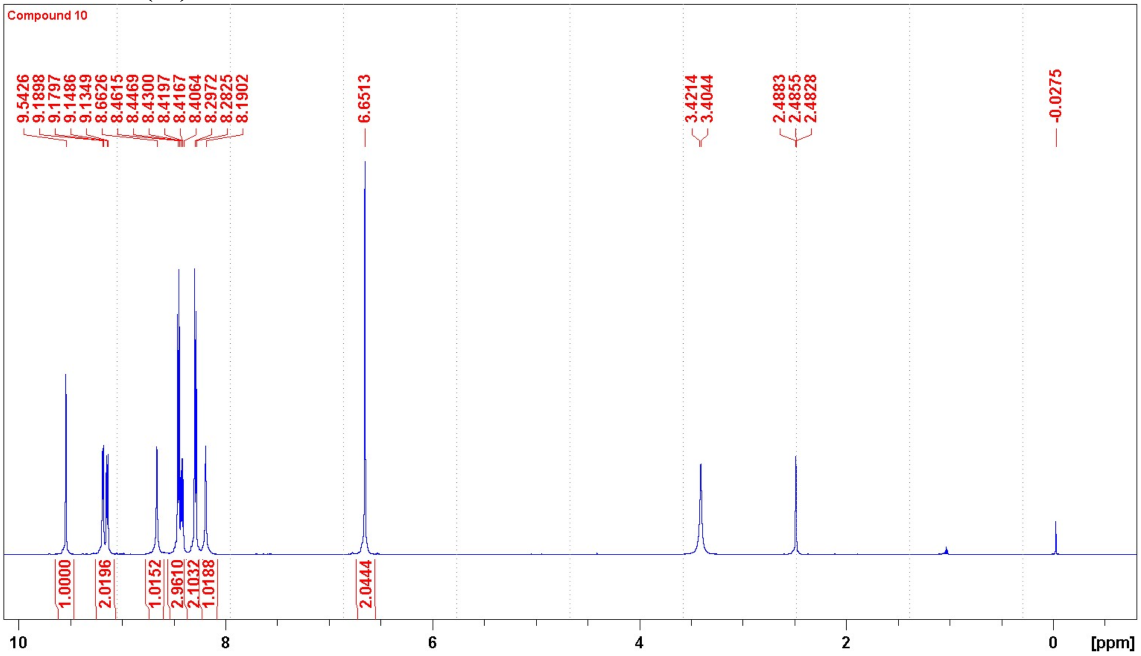

Figure S19. ^{1}H NMR (600 MHz, DMSO-d_6) spectrum of the compound **(10)**.

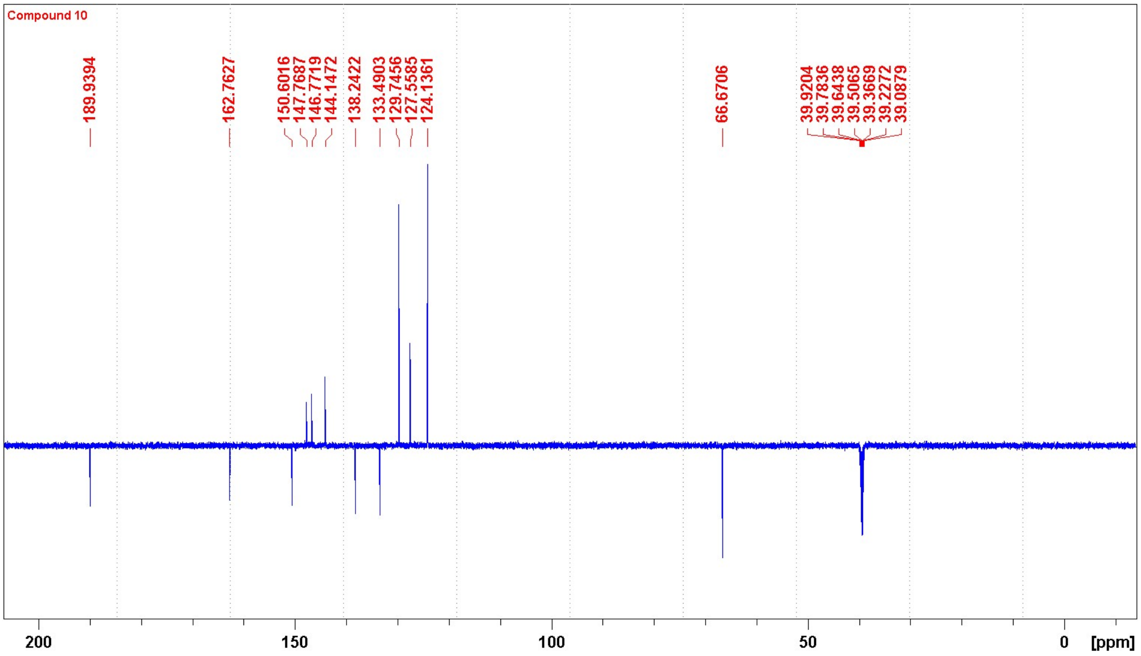

Figure S20. ^{13}C APT NMR (150 MHz, DMSO-d_6) spectrum of the compound **(10)**.

II) Copies of ^{1}H-^{1}H COSY NMR spectra of compounds **(2)** and **(8)**

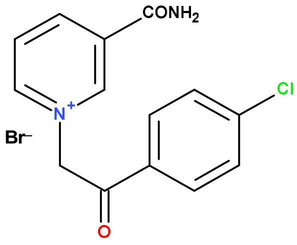

(2)

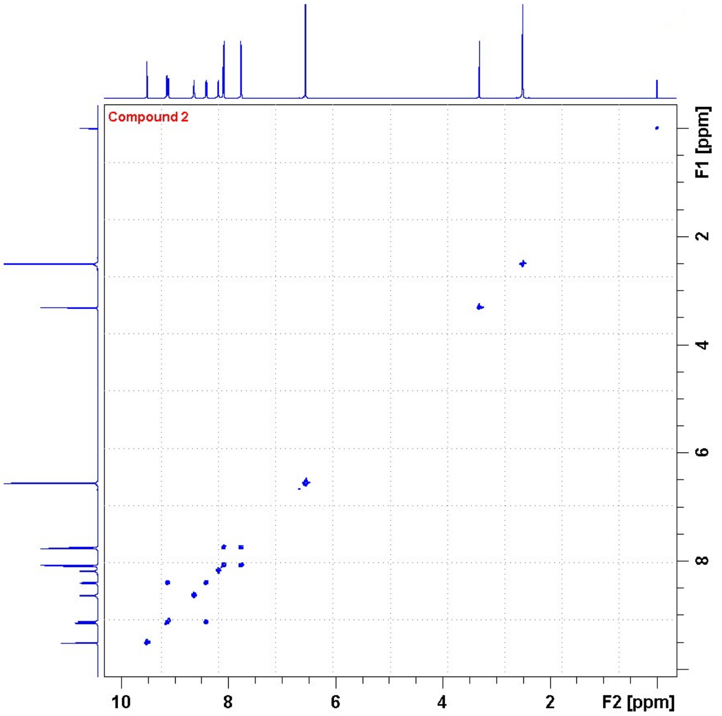

Figure S21. ^{1}H-^{1}H COSY NMR (600 MHz, DMSO-d_6) spectrum of the compound **(2)**. The one-dimensional ^{1}H NMR spectra are shown at the top and at the left-hand edge.

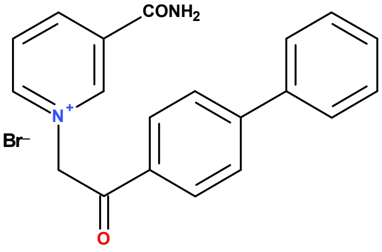

(8)

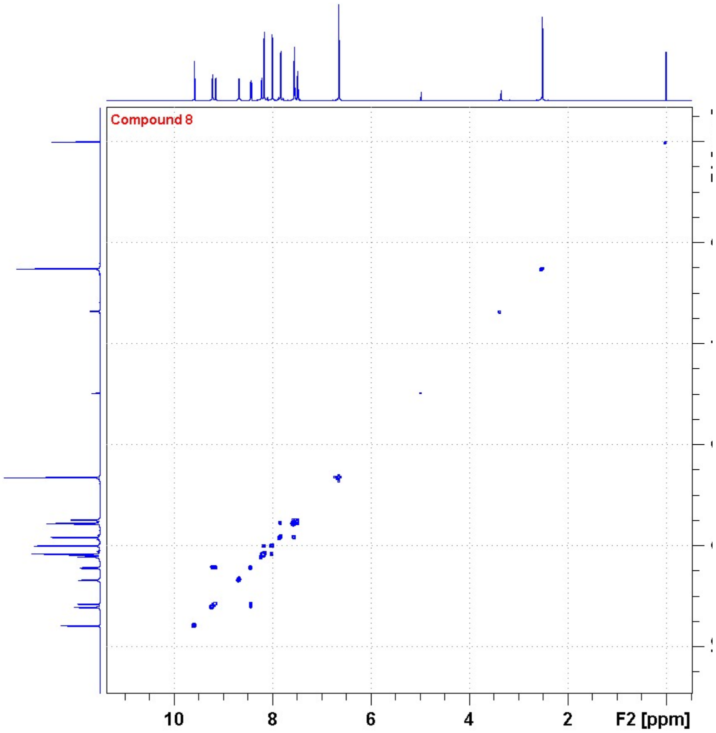

Figure S22. ^{1}H-^{1}H COSY NMR (600 MHz, DMSO-d_6) spectrum of the compound **(8)**. The one-dimensional ^{1}H NMR spectra are shown at the top and at the left-hand edge.

III) Copy of ^{1}H-^{13}C HMQC NMR spectrum of compound **(8)**

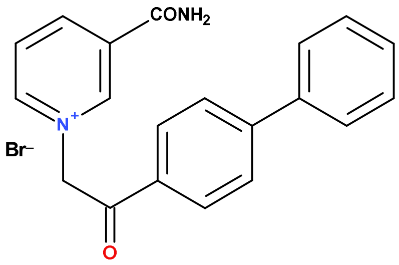

(8)

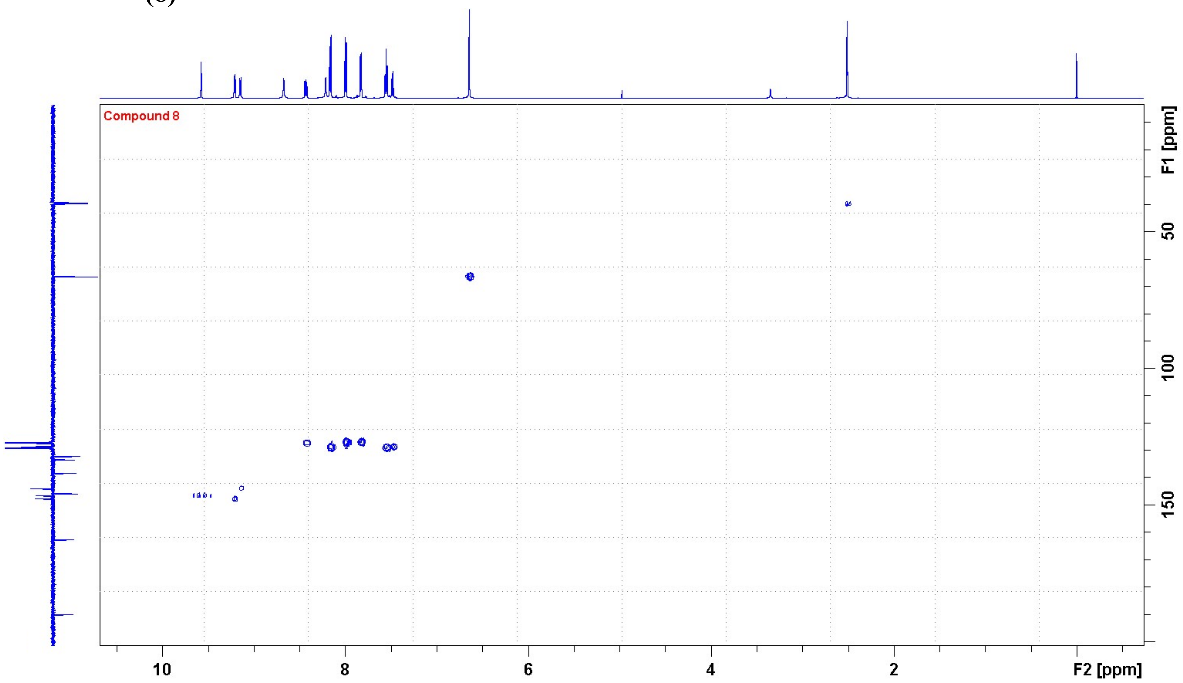

Figure S23. ^{1}H-^{13}C HMQC NMR spectrum of compound **(8)** in DMSO-d_6. The one-dimensional 600 MHz ^{1}H NMR spectrum is shown at the top edge and the 150 MHz ^{13}C NMR spectrum at the left-hand edge.

IV) Copies of ^{1}H-^{13}C HMBC NMR spectra of compounds **(2), (3)** and **(9)**

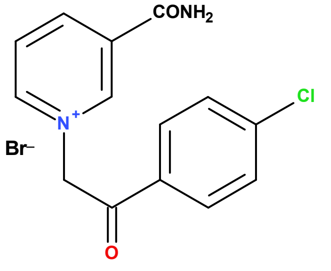

(2)

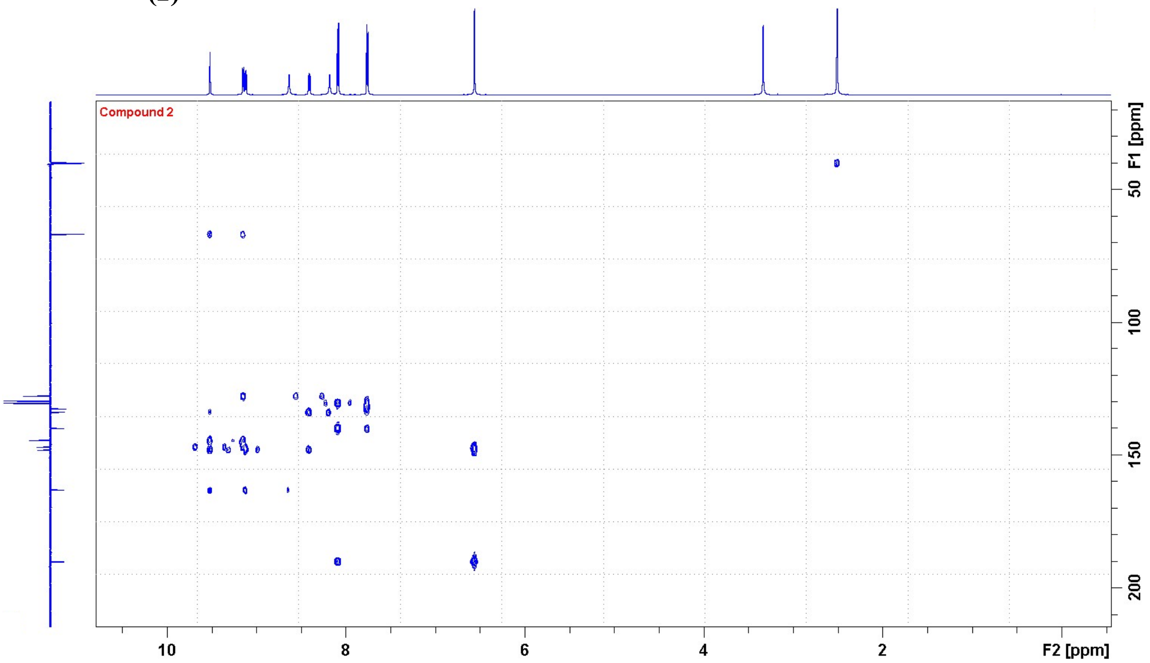

Figure S24. ^{1}H-^{13}C HMBC NMR spectrum of compound **(2)** in DMSO-d_6**.** The one-dimensional 600 MHz ^{1}H NMR spectrum is shown at the top edge and the 150 MHz ^{13}C NMR spectrum at the left-hand edge.

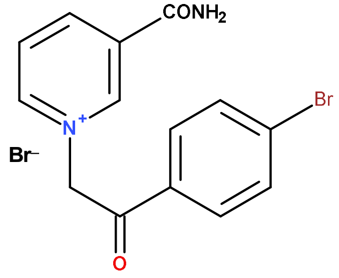

(3)

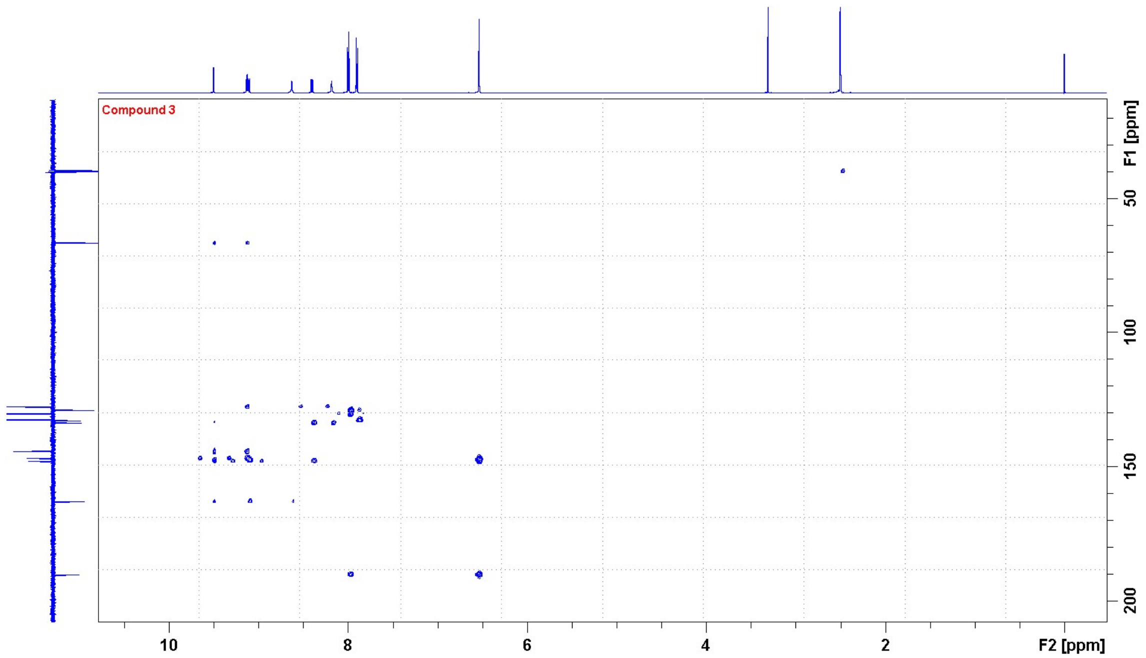

Figure S25. ${}^{1}H$-${}^{13}C$ HMBC NMR spectrum of compound **(3)** in DMSO-d_6**.** The one-dimensional 600 MHz ${}^{1}H$ NMR spectrum is shown at the top edge and the 150 MHz ${}^{13}C$ NMR spectrum at the left-hand edge.

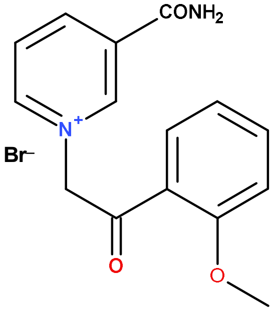

(9)

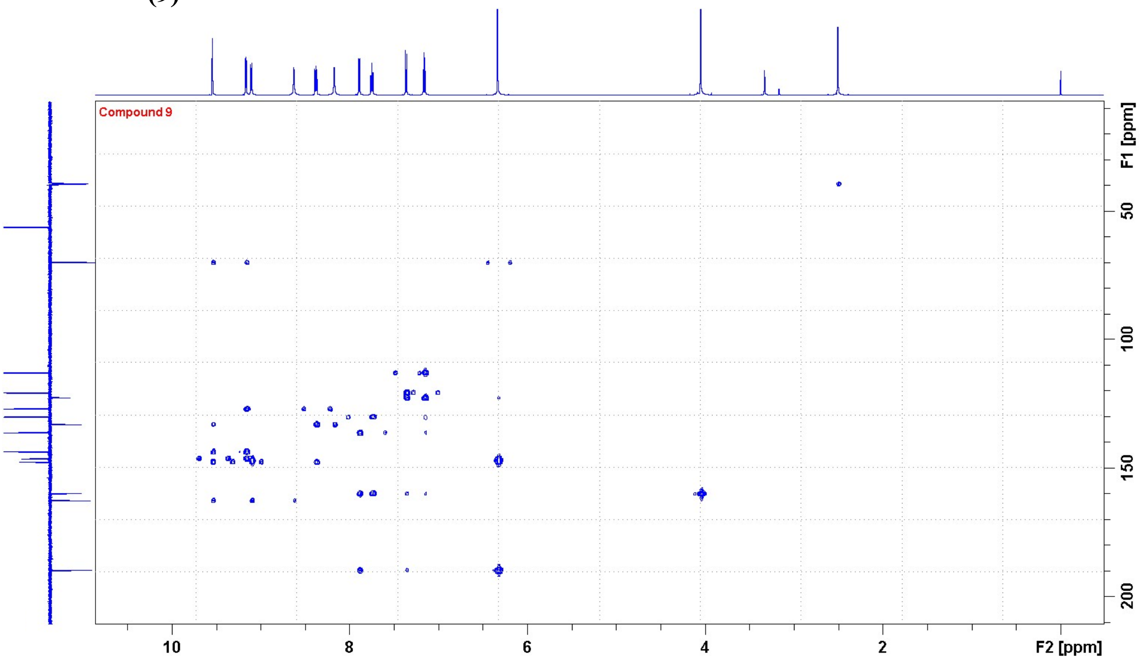

Figure S26. ^{1}H-^{13}C HMBC NMR spectrum of compound **(9)** in DMSO-d_6. The one-dimensional 600 MHz ^{1}H NMR spectrum is shown at the top edge and the 150 MHz ^{13}C NMR spectrum at the left-hand edge.
